# Plasma PAH-like AhR Bioactivity, Homocysteine, and Subclinical Vascular and Haematologic Phenotypes in the SPES Biomonitoring Substudy

**DOI:** 10.3390/ijms27219679

**Published:** 2026-10-29

**Authors:** Pellegrino Cerino, Elvira De Rosa, Maria Triassi, Paolo Montuori, Carlo Buonerba

**Affiliations:** 1 Department of Public Health, University of Naples “Federico II”, Via Sergio Pansini 5, 80131 Naples, Italy; 2 Department of Human Sciences and Quality of Life Promotion, San Raffaele University, 00166 Rome, Italy

**Keywords:** PAH-CALUX, aryl hydrocarbon receptor, effect-based biomonitoring, benzo[a]pyrene equivalents, homocysteine, blood pressure, haematology, SPES

## Abstract

Receptor-based bioassays measure mixture activity whose relationship with human physiology requires empirical assessment. We examined plasma polycyclic aromatic hydrocarbon (PAH)-like aryl hydrocarbon receptor bioactivity in the selected cross-sectional SPES biomonitoring substudy in Campania, Italy. Out of 598 assay records, 591 had quantified PAH-CALUX results and 7 were below individual limits of quantification (LOQs). Primary models related log2 lipid-normalised benzo[a]pyrene-equivalent bioactivity to homocysteine and recorded seated systolic blood pressure (SBP), adjusting for age, sex, body mass index, current smoking, and sampling cluster fixed effects, with municipality-clustered inference. Holm correction covered the two primary tests; Benjamini–Hochberg correction covered two secondary families. Participants comprised 295 women and 296 men aged 20–50 years from 63 municipalities. Per exposure doubling, homocysteine differed by −0.12% (95% confidence interval [CI], −0.76% to 0.52%; Holm-adjusted *p* = 0.699) and SBP by −0.37 mmHg (95% CI, −0.80 to 0.06; Holm-adjusted *p* = 0.183). None of the eighteen secondary outcomes met the within-family false discovery rate threshold. Additional post hoc Akins lipid normalisation and three below-LOQ substitution analyses yielded similar interpretations. These findings describe small estimated contemporaneous associations in a selected population. Uncertainty remains concerning cumulative exposure, temporal variability, and generalisability; equivalence and environmental safety cannot be inferred.

## 1. Introduction

Human biological samples contain constituents arising from environmental exposures, diet, medicines, and endogenous metabolism. The blood exposome analysis by Rappaport and colleagues illustrates the breadth of this internal chemical environment and the limitations of a small targeted panel [1]. Receptor-based assays add functional information by measuring the activity elicited by extracted mixtures. Relating that activity to clinical measurements is an important step in evaluating its relevance to human physiology within the integrated environmental and clinical framework of the Exposure Study on Susceptible People (SPES) [2].

Recombinant reporter systems couple receptor-responsive regulatory sequences to luciferase expression, translating cellular transcriptional activity into a measurable signal [3]. Calibration against a reference compound provides a common activity scale for mixtures whose active constituents may be incompletely characterised. The result depends on extraction, cellular uptake, metabolism, and assay duration [4]. Applications in drinking water sources illustrate how effect-based monitoring can complement chemical analysis [5]. This environmental evidence provides methodological context for the epidemiological assessment of human plasma bioactivity.

The aryl hydrocarbon receptor (AhR) has experimental links to vascular regulation and blood cell biology. Endothelial-cell-specific AhR deletion produced hypotension and altered angiotensin II responsiveness in mice [6], while pharmacological AhR antagonism promoted ex vivo expansion of human haematopoietic stem cells [7]. These findings motivate investigation of vascular and haematologic phenotypes alongside an exposure-oriented reporter assay. Their implications for human plasma mixtures require direct evaluation because receptor deletion, antagonism, and extract-induced reporter responses represent different biological perturbations.

The polycyclic aromatic hydrocarbon (PAH) chemically activated luciferase gene expression (CALUX) assay characterises PAH-like AhR bioactivity using stably transfected rat hepatoma H4IIe cells, four-hour exposure, and benzo[a]pyrene calibration [4]. In SPES, BioDetection Systems (BDS) applied this procedure to human plasma extracts. Results are expressed as benzo[a]pyrene equivalents (BaP-EQ), an operational measure of the extracted mixture’s activity under the assay conditions. Chemical identification of the contributing agonists and measurement of AhR signalling in participants’ tissues fall outside of the information supplied by this assay.

Human reporter assay studies provide relevant precedents. The Prospective Investigation of the Vasculature in Uppsala Seniors (PIVUS) related serum-induced AhR activity to circulating persistent pollutants, whereas the Brazilian Longitudinal Study of Adult Health (ELSA-Brasil) examined serum-induced activity in relation to incidence of type 2 diabetes [8,9]. Studies of urinary PAH metabolites provide chemically defined exposure comparisons [10]. Differences in matrices, preparation, cell systems, and exposure windows make these complementary lines of evidence. The present study assesses the relationship between short-incubation extracted plasma bioactivity and contemporaneous clinical phenotypes.

Homocysteine was selected as the primary biochemical phenotype because of its relationship to one carbon metabolism, its nutritional and renal determinants, and its use in environmental epidemiology [11]. Studies of ambient particles have reported associations that vary with smoking and participant susceptibility [12,13]. These observations provide a rationale for examining homocysteine in combustion-related exposure research. Its role here is a single, continuous biochemical measurement with high completeness and clear interpretation; specificity for AhR activation is limited.

Recorded seated systolic blood pressure (SBP) provided a complementary haemodynamic phenotype on a clinically familiar scale. Prospective evidence links usual blood pressure to vascular mortality [14], and a large Beijing study assessed blood pressure and homocysteine together in relation to long-term ambient pollution [15]. We investigated the two measurements as separate primary endpoints. Their selection did not imply a mediating pathway, and the interpretation of SBP was limited to the available single seated recording.

The secondary panel supplied physiological context. Diastolic blood pressure (DBP), pulse pressure, and heart rate extended the haemodynamic assessment; glucose and uric acid supplied metabolic context. The blood count family covered erythrocyte size and haemoglobinisation, red cell distribution width (RDW), leukocyte subsets, platelets, and derived ratios. Findings relating RDW to outcomes in heart failure and urinary PAH biomarkers to inflammatory blood measurements support the relevance of these domains [16,17]. Grouping related measurements into two secondary families allowed for complete reporting with separate control of multiplicity.

SPES provides paired biological samples, clinical records, and geographic information for this assessment. Related SPES analyses examined trace elements in relation to diet and reported health history [18,19]. For the present study, an internal plan prioritised homocysteine and SBP before association fitting using biological relevance, measurement quality, completeness, and temporal interpretability. Both were available for every participant with quantified PAH-CALUX. Vascular/metabolic and haematologic outcomes were assigned secondary roles. The objective was to estimate contemporaneous assay–phenotype associations per two-fold higher bioactivity in this selected population, with uncertainty and sensitivity to analytical choices explicitly reported.

## 2. Results

### 2.1. Participant Flow and Analytical Characteristics

**Figure 1:**
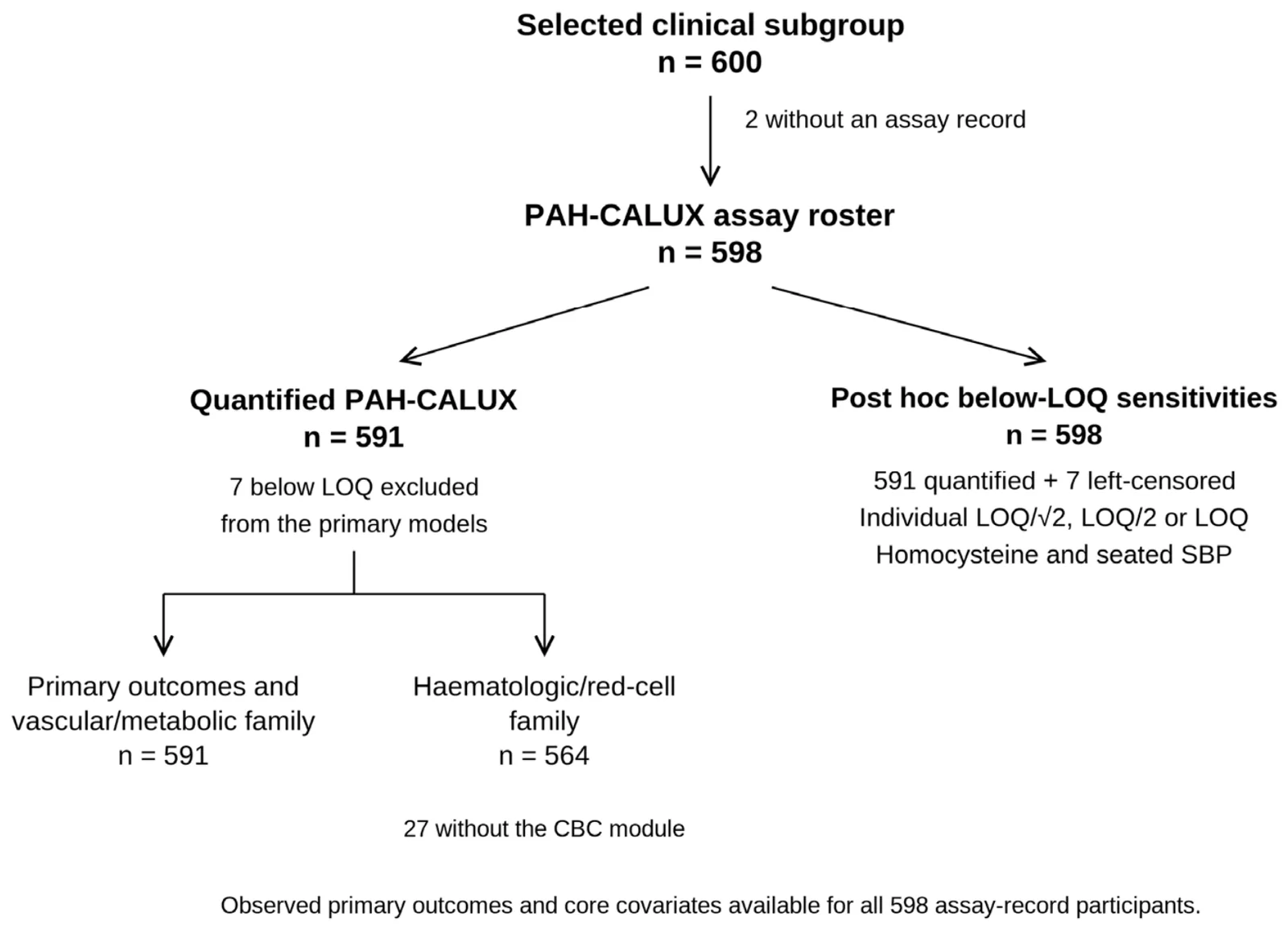
Participant flow and outcome-specific populations. Out of 600 selected participants, 598 had an assay record. The primary and vascular/metabolic analyses used 591 quantified PAH-CALUX results; the haematologic/red cell analyses used 564 participants with complete CBC measurements. All 598 assay record participants contributed to the post hoc below-LOQ substitution sensitivities for the two primary outcomes. The two participants without an assay record remained excluded.

The selected clinical subgroup contained 600 participants. Of these, 598 had an assay roster record and 2 had no CALUX assay record. Seven PAH-CALUX results were below their individual limits of quantification (LOQs), leaving 591 participants with positive quantified measurements for the primary analysis (Figure 1; Table S1). All 591 had homocysteine, recorded seated SBP, and the core covariates available; they also contributed to the five vascular/metabolic secondary models. The thirteen haematologic/red cell models included 564 participants, reflecting 27 participants without the complete blood count (CBC) module. The additional below-LOQ sensitivity models included all 598 participants with assay records (Section 2.5).

The analytical population comprised 295 women (49.9%) and 296 men (50.1%), with a median age of 34 years (interquartile range [IQR], 27–42) and median body mass index (BMI) of 24.44 kg/m^2^ (IQR, 22.42–27.34; Table 1). Recorded ages ranged from 20 to 50 years and BMI from 16.90 to 38.57 kg/m^2^. Participants came from 63 municipalities and 11 sampling cluster codes. Municipality sample sizes ranged from 1 to 34, with a median of 7; five municipalities contributed one participant. Low-, medium-, and high-impact areas contributed 119, 236, and 236 participants, respectively.

Two participants were recorded as current smokers. Antihypertensive treatment status was available for 589 participants: 37 had an explicit positive response, 5 had an explicit negative response, and 547 were classified as untreated from a negative hypertension history with a structurally skipped follow-up treatment item. Two questionnaire records were unavailable (Table S13). Physician-diagnosed hypertension was reported for 42 out of 589 participants with known history. Median homocysteine was 8.09 µmol/L (IQR, 7.20–9.30), and median recorded seated SBP was 120 mmHg (IQR, 110–130).

The seven participants with below-LOQ results were older (median 43 years; IQR, 33–45.5) and more often women (5/7) than the quantified group; median homocysteine was 9.19 µmol/L and median SBP was 120 mmHg (Table S11). CBC absence occurred in 11 out of 24 quantified participants in sampling cluster code 20 and 16 out of 34 in code 21, all in medium-impact areas. Median volume-based bioactivity was 39 ng BaP-EQ/mL among those without CBC data and 26 ng BaP-EQ/mL among those with CBC data (Table S12). The quantified subgroup’s area distribution also differed from the parent cohort: high-, medium-, and low-impact areas accounted for 39.9%, 39.9%, and 20.1% compared with 57.2%, 28.6%, and 14.3% in the parent study (Table S14).

### 2.2. PAH-CALUX Exposure and Measurement Support

Median laboratory-reported volume-based PAH-CALUX bioactivity was 27.00 ng benzo[a]pyrene equivalents (BaP-EQ)/mL (IQR, 6.80–89.00); median lipid-normalised bioactivity was 5150.98 ng BaP-EQ/g lipid (IQR, 1199.66–16,211.22). Volume-based values ranged from 0.26 to 7900 ng BaP-EQ/mL. Every quantified value was retained in the primary log2-exposure analysis without winsorisation.

Lipid-normalised exposure was reproduced from the laboratory-reported value and the historical clinical lipid component sum (Table S2). All quantified measurements, including the maximum of 7900 ng BaP-EQ/mL, were retained. BDS supplied additional dilution measurements for the plasma extract corresponding to the maximum result (Table S17). Figures S1 and S2 show the log2 distributions of lipid-normalised and volume-based bioactivity. Tables S3 and S3c provide the exposure and phenotype distributions.

### 2.3. Primary Associations

In the primary covariate-adjusted model, a two-fold higher lipid-normalised PAH-CALUX value was associated with a −0.12% difference in homocysteine (95% confidence interval [CI], −0.76% to 0.52%; raw *p* = 0.699; Holm-adjusted *p* = 0.699; Table 2). The design-adjusted estimate was −0.13% (95% CI, −0.76% to 0.51%).

For recorded seated SBP, the primary covariate-adjusted estimate was −0.37 mmHg per doubling (95% CI, −0.80 to 0.06; raw *p* = 0.091; Holm-adjusted *p* = 0.183). The design-adjusted estimate was −0.36 mmHg (95% CI, −0.84 to 0.13). Both primary intervals included zero and both Holm-adjusted tests exceeded 0.05. All primary and secondary confidence intervals are pointwise 95% intervals; multiplicity correction applies to the specified hypothesis tests.

### 2.4. Secondary Vascular/Metabolic and Haematologic Associations

None of the five vascular/metabolic outcomes met the within-family false discovery rate (FDR) threshold (Table 3). Estimates per exposure doubling were −0.18 mmHg for DBP, −0.19 mmHg for pulse pressure, −0.13 beats/min for heart rate, −0.016 mg/dL for uric acid, and 0.22 mg/dL for glucose. Every pointwise confidence interval included zero, and all five Benjamini–Hochberg (BH)-adjusted q values were 0.417.

The thirteen haematologic/red cell models likewise yielded no FDR-supported association. RDW differed by 0.017 percentage points per exposure doubling (95% CI, −0.018 to 0.052; q = 0.782). Estimated differences in mean corpuscular volume (MCV) and mean corpuscular haemoglobin (MCH) were −0.136 fL and −0.055 pg, respectively. Leukocyte and neutrophil counts differed by −0.70% and −0.74%, respectively. Their confidence intervals included the null; q values across the family ranged from 0.703 to 0.785. Table 3 reports the complete family, including haemoglobin, haematocrit, erythrocytes, mean corpuscular haemoglobin concentration (MCHC), lymphocytes, platelets, neutrophil to lymphocyte ratio (NLR), and platelet to lymphocyte ratio (PLR). Table S6 also provides the direct volume exposure sensitivity for both families.

### 2.5. Sensitivity and Exploratory Analyses

**Figure 2:**
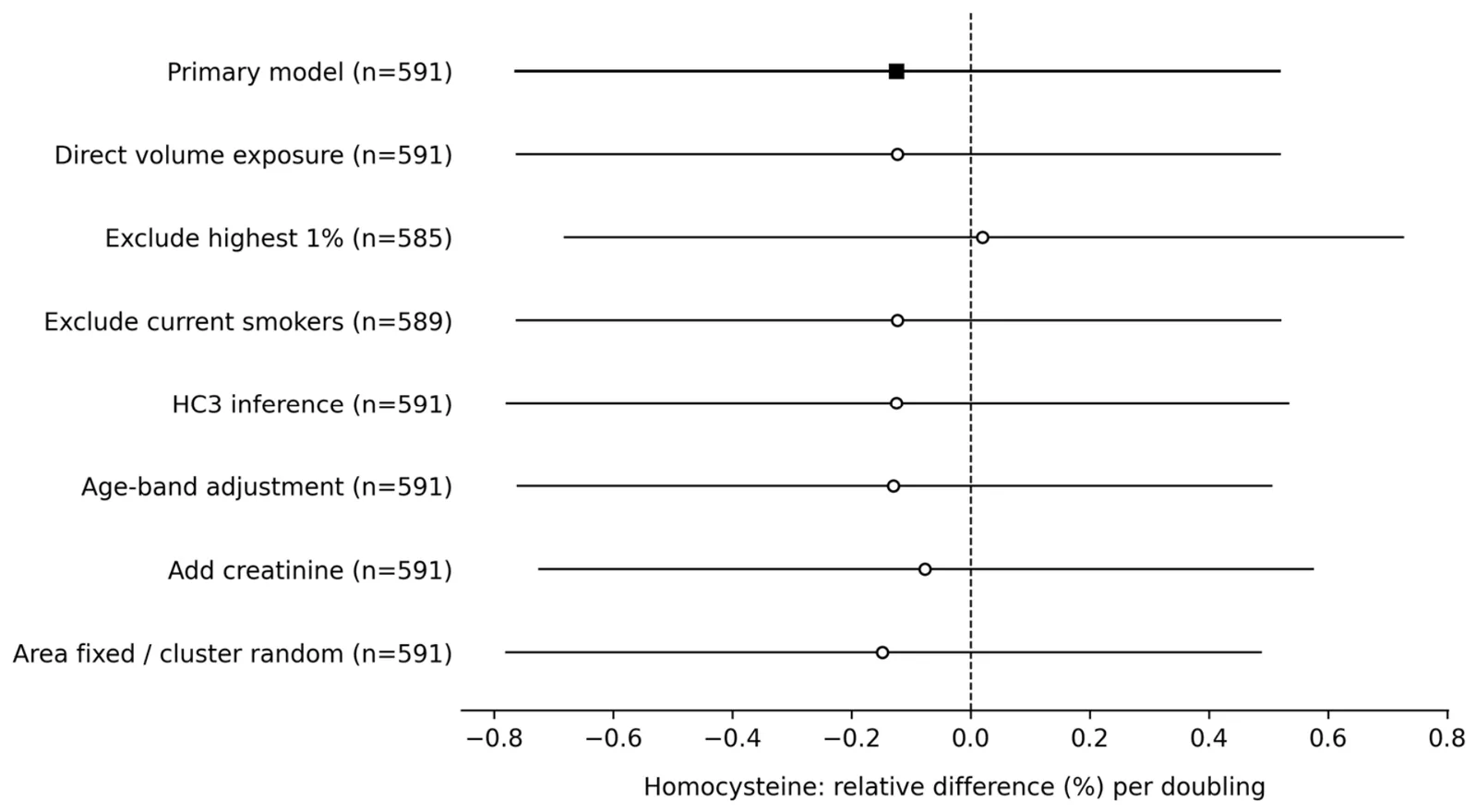
Homocysteine estimates in the primary model and specified sensitivity analyses. Effects are relative percentage differences in homocysteine per two-fold higher PAH-CALUX. Horizontal lines are pointwise 95% CIs. The filled square marks the primary Model 2 estimate; open circles denote sensitivities. Exposure is lipid-normalised except for the labelled volume-based analysis. Municipality-clustered intervals use G − 1 degrees of freedom; HC3 intervals use residual degrees of freedom (575); and the random intercept interval uses the nlme exposure term degrees of freedom (575). See Tables S5 and S8.

The homocysteine estimate varied little across the internally specified sensitivity analyses (Figure 2; Table S5). With direct volume exposure, it was −0.12% per doubling (95% CI, −0.76% to 0.52%). Excluding the highest 1% of the primary exposure yielded 585 participants and an estimate of 0.02% (95% CI, −0.68% to 0.73%). Excluding the two current smokers yielded 589 participants and an estimate of −0.12% (95% CI, −0.76% to 0.52%). Heteroscedasticity-consistent type 3 (HC3) inference and age band adjustment also gave estimates close to the primary value. Additional creatinine adjustment yielded −0.08% (95% CI, −0.72% to 0.57%).

**Figure 3:**
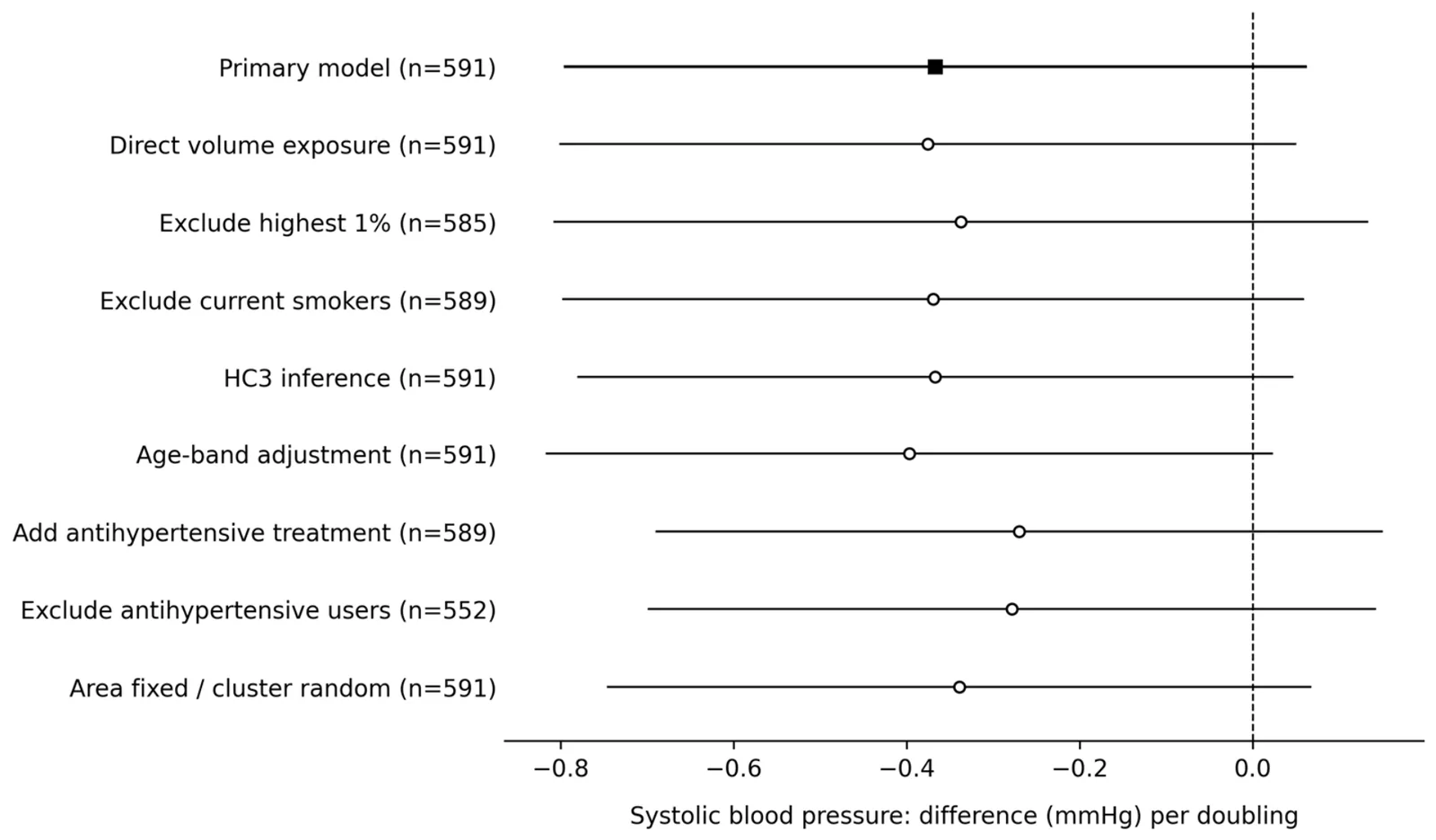
Systolic blood pressure estimates in the primary model and specified sensitivity analyses. Effects are adjusted mean differences in SBP, in mmHg, per two-fold higher PAH-CALUX. Symbols and interval conventions follow Figure 2. Treatment adjustment uses 589 participants with known status; the untreated-only analysis uses 552. See Tables S5 and S8 for model-specific estimates and inference.

The SBP point estimate remained modestly inverse in the corresponding analyses, with all pointwise intervals including zero (Figure 3; Table S5). Estimates were −0.38 mmHg with direct volume exposure, −0.34 mmHg after highest 1% exclusion, and −0.37 mmHg after excluding current smokers. Adjustment for antihypertensive treatment, using 589 participants with known treatment status, yielded −0.27 mmHg (95% CI, −0.69 to 0.15). Restriction to the 552 participants classified as untreated yielded −0.28 mmHg (95% CI, −0.70 to 0.14).

Restricted cubic spline tests gave nonlinear *p* values of 0.318 for homocysteine and 0.837 for SBP (global *p* = 0.513 and 0.124, respectively; Table S7b). In the exploratory pooled interaction models, homocysteine contrasts were +0.46% in women (95% CI, −0.52% to 1.44%) and −0.74% in men (−1.56% to 0.10%), with exposure by sex interaction *p* = 0.059. SBP contrasts were −0.64 mmHg in women (−1.08 to −0.20) and −0.08 mmHg in men (−0.82 to 0.66), with interaction *p* = 0.198 (Table S7). These pointwise contrasts provide exploratory estimates of heterogeneity.

The area-fixed, sampling cluster random intercept sensitivity gave estimates of −0.15% for homocysteine and −0.34 mmHg for SBP, with intervals including the null (Table S8). Maximum Cook’s distances were 0.045 and 0.041, and 32 and 25 observations, respectively, exceeded the descriptive 4/*n* threshold (Table S9). These flags did not determine primary exclusions.

Post hoc Akins sensitivity yielded a homocysteine difference of −0.129% per doubling (95% CI, −0.769% to 0.515%) and an SBP difference of −0.366 mmHg (−0.793 to 0.062). The historical component sum, Akins, and direct volume comparisons used the same 591 participants and 63 municipalities (Table S15).

All three post hoc below-LOQ scenarios included 598 participants and 63 municipalities (Table S18). Substitution by individual LOQ/√2, LOQ/2, and LOQ gave homocysteine estimates of −0.211%, −0.215%, and −0.207%, respectively, and SBP estimates of −0.323, −0.319, and −0.328 mmHg. All pointwise 95% intervals included zero. Relative to the primary estimates, homocysteine differences became 0.08–0.09 percentage points more negative and SBP differences moved 0.04–0.05 mmHg towards zero.

## 3. Discussion

In this selected SPES substudy, the estimated contemporaneous associations of plasma PAH-like AhR bioactivity with homocysteine and recorded seated SBP were small. Per exposure doubling, homocysteine differed by −0.12% (95% CI, −0.76% to 0.52%) and SBP by −0.37 mmHg (−0.80 to 0.06). Neither primary test met the Holm-adjusted criterion, and none of eighteen secondary outcomes met its within-family FDR threshold. The intervals quantify uncertainty around model-based contrasts in the measured phenotypes. They remain subject to selection, measurement error, and residual confounding and provide no basis for inferring equivalence or environmental safety [20].

Homocysteine and SBP were prioritised for complementary biological domains, continuous measurement, and complete availability. Homocysteine reflects nutritional and renal determinants of one-carbon metabolism [11]. Reports of particulate matter associations have varied with smoking and participant characteristics [12,13], while urinary PAH research has found null-compatible adjusted homocysteine and inflammatory marker comparisons among nonsmokers [10]. Studies of ambient pollution have also considered homocysteine and blood pressure jointly [15]. These findings support the choice of domains while illustrating how population and exposure definitions affect interpretation. The HOPE-2 trial further demonstrates the distinction between changing a biochemical phenotype and improving a clinical cardiovascular outcome [21].

The SBP estimate was modestly inverse and remained compatible with zero across the planned sensitivity analyses. Urinary PAH studies have reported differing blood pressure associations in community and occupational settings [22,23]. Their exposure matrices, populations, and measurement schedules differ from this plasma extract analysis. In SPES, treatment adjustment and restriction to those classified as untreated gave slightly attenuated estimates. The available outcome was one recorded seated reading; evidence on repeated measurement usual blood pressure and vascular mortality concerns a different level of measurement reliability and follow-up [14]. An independent random error in a dependent variable chiefly reduces precision, whereas systematic or exposure-related errors may bias an association.

PAH-CALUX supplies molecularly informed exposure characterisation through the transcriptional response of an AhR-responsive reporter system [3,4]. Calibration expresses the recovered mixture activity relative to BaP under controlled in vitro conditions. The chemical contributors and the extent of AhR activation in participant tissues remain unresolved. Experimental vascular and haematopoietic AhR studies provide biological context [6,7], while PIVUS and ELSA-Brasil demonstrate ways to study serum reporter activity alongside chemical biomarkers or incident disease [8,9]. Environmental effect-based monitoring provides another application of this complementary approach [5]. The contribution of SPES is a defined extracted plasma assay–phenotype comparison in selected younger adults with a complete outcome panel and explicit uncertainty.

The blood count family provided secondary physiological context. Prior urinary biomarker studies linked PAHs to inflammatory blood measurements [17], and RDW has prognostic associations in established heart failure [16]. These settings provide limited guidance for the direction or magnitude of peripheral count associations in the SPES subgroup. The thirteen haematologic models involved 564 participants because the entire CBC module was unavailable for 27. All missing records came from two sampling clusters, and the CBC missing group had higher median bioactivity. Table S12 makes this selection visible. Geographic adjustment may account for part of the observed pattern, while the relationship between missingness and unobserved blood counts remains uncertain. The complete case results require this qualification.

Lipid handling was a further analytical consideration because circulating lipids can be related to both measured bioactivity and health [24]. The primary exposure preserved the historical clinical component sum. Direct volume and additional Akins-normalised analyses produced estimates close to the primary results, supporting robustness under those alternative exposure definitions. The seven below-LOQ results were addressed separately in three post hoc substitution scenarios using all 598 assay record participants. Their inclusion produced small changes in the primary estimates and retained the original interpretation. These comparisons concern explicit assumptions about unquantified activity; their conditional intervals omit uncertainty in the true sub-LOQ values, and the LOQ-boundary scenario is not a worst case coefficient bound.

The expanded assay documentation provides recovery, reference control, quantification limit, and dilution information from the original laboratory. Additional dilution measurements concern the plasma extract with the maximum reported bioactivity. Variation among dilution-corrected estimates introduces uncertainty around its exact magnitude, although excluding the highest 1% of values yielded similar findings for both primary outcomes (Table S5). Published assay development precision is reported separately from SPES control performance. Complete campaign-specific precision estimates, calibration acceptance criteria, and sample-specific cytotoxicity/interference data were unavailable. Laboratory measurement error and within-person exposure variability may attenuate associations, although their magnitude and direction cannot be quantified for these data.

The cross-sectional design is central to interpretation. Bioactivity and clinical phenotypes were obtained from the enrolment encounter, with one measurement of each. The four-hour exposure denotes reporter cell incubation, and a single in vivo half-life cannot be assigned to the extracted mixture’s bioactivity. Temporal order, within-person persistence, and the exposure window relevant to vascular or biochemical change remain uncertain. Routine homocysteine records also lacked complete assay platform and preanalytical traceability. The documented processing of stored CALUX plasma is kept separate from the routine clinical workflow. Repeated sampling would help distinguish persistent between-person variation from transient biological and analytical variation.

Residual confounding is particularly relevant to homocysteine. Folate and vitamin B12 were unavailable, and the primary models omitted dietary, alcohol, occupational, socioeconomic, and detailed medication variables. Creatinine adjustment changed the homocysteine estimate little and addressed only one renal function marker. The near absence of current smoking limited information available to the binary smoking term; excluding the two recorded smokers produced similar estimates. These checks assess dependence on specified model choices. Remaining confounding could mask, exaggerate, or change the direction of an association.

Selection limits generalisability. The intensive biomonitoring subgroup was balanced on design characteristics and allocated across environmental impact areas in a different proportion from the parent cohort. Recorded smokers, treated participants, and BMI values exceeding the intended restriction were retained; the available records could not reconstruct the reasons for individual departures. Table S14 describes the geographic and demographic comparison with the parent study, which includes the subgroup itself. The original primary analysis further concerns quantifiable bioactivity, with the additional censored value scenarios extending the assessment under explicit assumptions. The observed age range and recruitment criteria constrain extrapolation to older, occupationally exposed, or otherwise different populations.

Internal specification, exact linkage, complete primary outcome information, full reporting of both secondary families, and preservation of the original inference are strengths. Complete reporting reduces selective emphasis on individual results, an established concern in empirical research [25]. The exploratory sex contrasts remain hypothesis-generating, including the homocysteine interaction *p* value of 0.059; differing within-sex interval inclusion of zero does not establish heterogeneity. Shared participants and clinical measurements across related SPES papers also require recognition when interpreting the programme’s evidence [18,19,26]. Future studies should combine repeated bioactivity and clinical measurements with nutritional biomarkers, treatment and exposure histories, and targeted chemistry at compatible times. Such data would support assessment of temporal and mechanistic questions beyond the contemporaneous associations estimated here.

## 4. Materials and Methods

### 4.1. Design, Setting, and Population

This secondary cross-sectional analysis used the SPES CALUX biomonitoring substudy in Campania, Italy. The parent programme enrolled 4205 participants during 2016–2017 and combined environmental geostratification, questionnaires, clinical assessment, and biospecimen collection [2]. A 600-person subgroup was selected for extended biomonitoring, balancing age, sex, and BMI and targeting a 2:2:1 distribution across high-, medium-, and low-impact areas to increase geographic contrast [27]. Intended restrictions included no current smoking, no alcohol abuse, no chronic disease medication, and exclusion of higher obesity classes; the study-specific documentation also described exclusion of participants positive for human immunodeficiency virus (HIV), hepatitis B virus (HBV), or hepatitis C virus (HCV) (BDS, direct communication). The observed analytical age range was 20–50 years.

The primary population included participants with positive quantified PAH-CALUX and the required observed outcomes and covariates. Two selected participants lacked an assay record, and seven results were below individual LOQs, leaving 591 participants. Sample size was determined by the archived substudy and assay availability. Recorded smokers, antihypertensive users, and participants with BMI values exceeding the intended restriction were retained. The available records could not establish the reason for each departure from the intended selection criteria. Additional post hoc sensitivities evaluated inclusion of the seven censored results; the original population remained primary.

### 4.2. Participant Linkage and Analysis Specification

CALUX and clinical records were linked by exact blood kit code. An upstream laboratory file containing both kit code and participant identifier supplied a one-to-one crosswalk checked against demographics and independently identifier-keyed clinical report form records. All 600 subgroup kit codes mapped to unique participants. Seated blood pressure was recovered from the original clinical record field. The subgroup BMI field was retained after source comparison.

The endpoint hierarchy and statistical analysis plan were frozen internally on 2 September 2026 before the initial association calculation release. The corrected R implementation was executed on 4 September 2026. Source files, code, and results were versioned and checksummed. The analysis was internally specified and had no external preregistration. Later implementation and reporting corrections preserved the primary exposure, outcomes, and models. An Akins lipid denominator amendment was prepared on 21 October and executed on 22 October 2026. A separate amendment authorising the three below-LOQ substitution scenarios was approved and executed on 24 October 2026. Both additions followed knowledge of the original results and reviewer comments and are reported as post hoc, alongside the revision-stage descriptive comparisons (Supplementary Methods S1, S9 and S10).

### 4.3. Plasma PAH-CALUX Measurement

PAH-CALUX analyses were performed by BioDetection Systems (BDS), Amsterdam, the Netherlands. Following the official SPES sample processing procedure, blood collected into lithium heparin tubes was centrifuged at 2500× *g* for 10 min at +4 °C; separated plasma was aliquoted and stored at −80 °C [27]. Plasma was shipped frozen on dry ice to BDS. Aliquots of 0.5 g of human plasma were extracted twice with 5 mL methyl-tert-butyl ether. Pooled extracts were evaporated under nitrogen and reconstituted in 25 µL dimethyl sulfoxide (DMSO). Mean BaP extraction and cleanup recovery was 77%. Serial extract dilutions were tested in technical triplicate in stably transfected H4IIe rat hepatoma reporter cells for four hours at 0.8% DMSO. Each 96-well plate included a complete BaP calibration curve; luciferase-dependent luminescence was interpolated using GraphPad Prism 5.03 (GraphPad Software, Inc., San Diego, CA, USA) and reported as ng BaP-EQ/mL plasma (BDS, direct communication).

Procedure blanks and BaP-spiked human reference plasma accompanied the analytical series. Mean reference plasma bioactivity was approximately 23 ng BaP-EQ/mL. High-response extracts underwent additional dilution to obtain responses within the linear assay range. BDS supplied additional dilution measurements for the plasma extract corresponding to the maximum reported activity. Seven measurements were below their individual LOQs of 0.14–0.41 ng BaP-EQ/mL and were excluded from the primary models. All 591 quantified measurements were retained. Supplementary Methods S3 and Tables S16 and S17 report quantitative quality control (QC), dilution checks, and the limits of the available analytical records.

### 4.4. Exposure Units and Lipid Derivation

The primary exposure retained the historical clinical lipid component sum recorded in the Istituto Zooprofilattico Sperimentale del Mezzogiorno (IZSM) dataset. Total cholesterol (TC), triglycerides (TG), and phospholipids (PL) were measured in serum and recorded in mg/dL. Recalculation reproduced the archived IZSM-labelled lipid field across all 598 assay records. The conversion was

Lipid percentage (% *w*/*v*; g/100 mL) = (TC + TG + PL)/1000.

PAH-CALUX (ng BaP-EQ/g estimated lipid) = PAH-CALUX (ng BaP-EQ/mL)/(lipid percentage/100),

with lipid mass expressed in g/mL.

The component sum maintained continuity with the original SPES exposure definition. It supplies an operational lipid denominator whose influence was assessed through direct volume and post hoc Akins sensitivities. The original laboratory-reported per-volume bioactivity was retained at full precision without an additional density or recovery correction. The primary exposure was log2-transformed, giving a per-doubling contrast, and analysed without winsorisation. The primary and internally specified sensitivities used quantified exposures only. Section 4.7 describes the separate post hoc substitutions.

### 4.5. Outcomes and Covariates

Primary outcomes were homocysteine (µmol/L), analysed on the natural-log scale, and recorded seated SBP (mmHg), analysed in its native units. Routine biochemical and haematologic values came from linked clinical laboratory records. Questionnaire completion, clinical assessment, and blood collection belonged to the same enrolment encounter. Published field procedures describe an instruction to fast for at least eight hours and a staff check before venepuncture [18]. Individual fasting duration, clock time, and exact within-visit intervals were unavailable. The official report describes fresh material allocated to routine biochemical and virological analyses within three hours of collection, separately from the aliquots retained for CALUX [27]. The retained records did not establish the homocysteine assay platform, assay-specific processing interval, or specimen to method traceability for every result.

The seated pressure field recorded SBP, DBP, heart rate, and oxygen saturation, in that order. One seated SBP recording was available per participant. Device model, resting interval, cuff selection procedure, repeat measurements, and timing relative to venepuncture were unavailable in the retained records. Interpretation therefore concerns the recorded seated value.

The secondary vascular/metabolic family contained DBP, pulse pressure, heart rate, uric acid, and glucose. Pulse pressure was SBP minus DBP. The haematologic/red cell family contained RDW, haemoglobin, haematocrit, erythrocyte count, MCV, MCH, MCHC, leukocytes, neutrophils, lymphocytes, platelets, NLR, and PLR. Counts and ratio units were harmonised before derivation: NLR was neutrophils/lymphocytes and PLR was (platelets/1000)/lymphocytes because the source platelet and lymphocyte units differed. Erythrocytes were displayed in millions/µL. Leukocytes, neutrophils, lymphocytes, platelets, NLR, and PLR were natural-log-transformed; the remaining secondary outcomes were analysed in their native units (Table S4).

Core covariates were age, sex, BMI, current smoking, and sampling cluster code. The approved linkage supplied municipality and antihypertensive treatment information. The treatment question followed the question on physician-diagnosed hypertension. An explicit negative history with a blank follow-up was classified as a structural skip and coded as untreated; missing history or an unavailable questionnaire remained unknown. This yielded 37 explicit positive responses, 5 explicit negatives, 547 derived negatives, and 2 unknown records (Table S13). Creatinine was available for homocysteine sensitivity. Folate and vitamin B12 measurements were unavailable for this analysis. Diet, alcohol, occupation, socioeconomic circumstances, and detailed medication information were outside of the frozen primary adjustment set.

### 4.6. Primary Estimation, Multiplicity, and Missing Data

Unweighted ordinary least-squares Model 1 included log2 exposure, age centred at 35 years and scaled per 10 years, sex, and fixed effects for 11 sampling-cluster codes. Model 2, the primary model, additionally included BMI centred at the analytical mean and current smoking. Female sex and sampling cluster code 9 were references. Environmental impact area was not entered simultaneously with unrestricted sampling cluster effects.

Municipality-clustered sandwich covariance [28] used the finite-sample multiplier [G/(G − 1)] × [(N − 1)/(N − K)], where G was the municipality count, N the model sample size, and K the fitted matrix rank. Primary outcome models used N = 591 and G = 63; K = 14 for Model 1 and K = 16 for Model 2. Primary tests and pointwise 95% CIs used a t reference with G − 1 = 62 degrees of freedom. For log-transformed outcomes, 100[exp(β) − 1] and the corresponding transformed interval limits express relative differences in conditional geometric means per exposure doubling. Native-scale coefficients express adjusted mean differences.

Two-sided inference used a 0.05 threshold. Holm correction covered the two Model 2 primary exposure tests [29]. Benjamini–Hochberg correction was applied separately within the five vascular/metabolic and thirteen haematologic/red-cell outcomes [30]. Volume-based sensitivities retained their labelled within-family corrections. Confidence intervals are pointwise. The original models used observed quantified exposures and complete outcomes and covariates: 591 participants for the primary and vascular/metabolic models and 564 for the haematologic models. The post hoc below-LOQ scenarios described in Section 4.7 were the sole exception to the original exposure substitution rule. Outcomes, covariates, and missing assay records were never imputed.

### 4.7. Sensitivities, Diagnostics, and Reproducibility

Sensitivity analyses used volume-based bioactivity, excluded observations above the type-7 99th percentile of the primary exposure, excluded current smokers, used HC3 covariance, and substituted age categories of <30, 30–39, and ≥40 years. The smoking term was omitted in the all-non-smoker subset. Homocysteine was additionally adjusted for creatinine. SBP models additionally adjusted for antihypertensive treatment or were restricted to untreated participants. HC3 tests and CIs used residual degrees of freedom, N − K = 575 in these fits.

Exploratory models added exposure × sex or a restricted cubic spline with knots at the 10th, 50th, and 90th percentiles of log2 exposure. Overall spline inference used a two-degree-of-freedom robust F test with municipality-based denominator degrees of freedom; the nonlinear component used a *t* test. Sex-specific estimates were linear contrasts from the pooled model. Geographic sensitivity used impact area fixed effects and a sampling cluster random intercept fitted by maximum likelihood in nlme; exposure term inference used the degrees of freedom returned by nlme (575). Cook’s distance and externally studentised residuals were evaluated without outcome-dependent primary exclusions.

The additional Akins sensitivity used measured serum free cholesterol (FC), TC, TG, and PL in the total lipid (TL) equation TL = 1.677(TC − FC) + FC + TG + PL, with all components in mg/dL [31]. Volume-based PAH-CALUX was divided by TL/100,000 (g/mL) and log2-transformed. Both primary outcome models retained the original covariates and municipality-clustered inference for the same 591 participants. Supplementary Methods S9 describes this post hoc analysis.

Three reviewer-requested below-LOQ sensitivities assigned each of the seven censored results its individual LOQ/√2, LOQ/2 or LOQ on the volume-based scale and then applied the original component sum denominator and log2 transformation. All quantified measurements were unchanged. Each scenario fitted the two original primary models, with the original BMI-centring reference and factor coding, among 598 participants with complete data. Intervals used municipality-clustered covariance and t62 inference and were conditional on the assigned values. The LOQ boundary is an assumed exposure limit; it does not bound the regression coefficient. All scenarios were reported as post hoc, with the original primary Holm correction unchanged (Supplementary Methods S10).

Original analyses were performed in R 4.5.1 (R Foundation for Statistical Computing, Vienna, Austria) on 64-bit Windows. Both post hoc sensitivity analyses used R 4.6.1 and reproduced the original primary estimates before fitting the additional models. Reporting follows the cross-sectional Strengthening the Reporting of Observational Studies in Epidemiology (STROBE) recommendations and the Molecular Epidemiology extension (STROBE-ME) [32,33]. Model definitions, software versions, aggregate estimates, and diagnostics are reported in the Supplementary Materials; data-free code and full-precision tables are supplied separately in Non-published Material.zip. Related SPES trace element work and companion dioxin-responsive (DR)-CALUX, oestrogen receptor alpha (ERα)-CALUX, and chemical polychlorinated dibenzo-p-dioxin/dibenzofuran (PCDD/F)–polychlorinated biphenyl (PCB) analyses share participants and some clinical measurements [18,19,26]. Each addresses a distinct exposure question; multiplicity control here applies to the specified PAH-CALUX families.

### 4.8. Generative AI Assistance

During preparation of this work, the authors used ChatGPT (OpenAI; GPT-5.6 Pro) and Claude Opus 5 (Anthropic) for editorial assistance and code generation. Participant measurements originated from archived study sources, and all statistical estimates were calculated in R. The authors reviewed and edited the AI-assisted material and take full responsibility for the specifications, code, results, interpretation, and final manuscript.

## 5. Conclusions

In selected SPES adults with an observed age range of 20–50 years, estimated contemporaneous associations of plasma PAH-like AhR bioactivity with homocysteine and recorded seated SBP were small, with pointwise confidence intervals including zero. No secondary outcome met its within-family FDR threshold. Additional Akins and below-LOQ sensitivities supported the original interpretation under the specified analytical assumptions. These cross-sectional findings apply to the measured phenotypes and selected population. Equivalence and environmental safety remain unestablished. Repeated paired bioactivity and clinical measurements, nutritional biomarkers, and complementary chemical characterisation are needed to assess temporal and clinical relevance.

## Figures and Tables

**Table 1:** Characteristics of the quantified PAH-CALUX analytical population.

Characteristic	Unit	*n*	Missing	Summary
Female sex	*n* (%)	591	0	295 (49.9%)
Male sex	*n* (%)	591	0	296 (50.1%)
Age 20–29 years	*n* (%)	591	0	197 (33.3%)
Age 30–39 years	*n* (%)	591	0	198 (33.5%)
Age ≥ 40 years (maximum 50)	*n* (%)	591	0	196 (33.2%)
Age	years	591	0	34.0 (27.0–42.0)
Body mass index	kg/m^2^	591	0	24.44 (22.42–27.34)
Environmental impact area: low	*n* (%)	591	0	119 (20.1%)
Environmental impact area: medium	*n* (%)	591	0	236 (39.9%)
Environmental impact area: high	*n* (%)	591	0	236 (39.9%)
Current smoking	*n* (%)	591	0	2 (0.3%)
Reported antihypertensive use	*n* (%)	589	2	37 (6.3%)
Reported physician-diagnosed hypertension	*n* (%)	589	2	42 (7.1%)
Complete CBC/red cell panel	*n* (%)	591	0	564 (95.4%)
PAH-CALUX, volume-based	ng BaP-EQ/mL	591	0	27.00 (6.80–89.00)
PAH-CALUX, IZSM lipid-normalised	ng BaP-EQ/g lipid	591	0	5150.98 (1199.66–16,211.22)
IZSM lipid component sum	%	591	0	0.534 (0.475–0.603)
Homocysteine	µmol/L	591	0	8.09 (7.20–9.30)
Seated systolic blood pressure	mmHg	591	0	120.0 (110.0–130.0)
Seated diastolic blood pressure	mmHg	591	0	80.0 (70.0–80.0)
Heart rate	beats/min	591	0	73.0 (66.0–80.0)
Creatinine	mg/dL	591	0	0.80 (0.70–0.90)
Glucose	mg/dL	591	0	77.0 (72.0–84.0)
Uric acid	mg/dL	591	0	4.70 (3.90–5.70)
Red cell distribution width	%	564	27	12.04 (11.65–12.60)

Values are *n* (%) or median (IQR). Percentages use the available denominator. CBC completeness denotes availability of the entire panel; individual CBC measures have *n* = 564 and 27 missing. The population is a selected biomonitoring subgroup. BaP-EQ, benzo[a]pyrene equivalents; CBC, complete blood count; IZSM, Istituto Zooprofilattico Sperimentale del Mezzogiorno. Lipid percentage is % *w*/*v*. Assay values retain the laboratory-reported per-volume scale before the specified lipid normalisation.

**Table 2:** Primary associations per two-fold higher IZSM lipid-normalised PAH-CALUX bioactivity.

Endpoint/Model	*n*	Difference	95% CI	Raw *p*	Holm *p*
Homocysteine—M1	591	−0.13%	−0.76 to 0.51	0.694	—
Homocysteine—M2	591	−0.12%	−0.76 to 0.52	0.699	0.699
Seated systolic blood pressure—M1	591	−0.36 mmHg	−0.84 to 0.13	0.144	—
Seated systolic blood pressure—M2	591	−0.37 mmHg	−0.80 to 0.06	0.091	0.183

Model 1: exposure, (age − 35)/10, sex and sampling cluster fixed effects. Model 2 adds BMI centred at the analytical mean and current smoking. Covariance is municipality-clustered, with the finite sample correction and t62 inference described in the Methods. The 95% CIs are pointwise, not multiplicity-adjusted. Holm correction applies only to the two Model 2 exposure tests. Homocysteine effects are relative percentage differences in conditional geometric means; SBP effects are adjusted mean differences in mmHg. Both models use 63 municipalities; M1 has 14 fitted columns and M2 has 16, including the intercept.

**Table 3:** Prespecified secondary associations per two-fold higher lipid-normalised PAH-CALUX.

Outcome	*n*	Difference	95% CI	Raw *p*	BH q
**Vascular/metabolic**					
Seated diastolic blood pressure	591	−0.181 mmHg	−0.508 to 0.146	0.272	0.417
Pulse pressure	591	−0.186 mmHg	−0.615 to 0.243	0.390	0.417
Heart rate	591	−0.134 beats/min	−0.462 to 0.194	0.417	0.417
Uric acid	591	−0.016 mg/dL	−0.047 to 0.014	0.292	0.417
Glucose	591	0.219 mg/dL	−0.070 to 0.508	0.135	0.417
**Haematologic/red cell**					
Red cell distribution width	564	0.017 pp	−0.018 to 0.052	0.334	0.782
Haemoglobin	564	−0.011 g/dL	−0.059 to 0.036	0.637	0.782
Haematocrit	564	−0.025 pp	−0.165 to 0.115	0.722	0.782
Erythrocyte count	564	0.006 million/µL	−0.008 to 0.021	0.387	0.782
Mean corpuscular volume	564	−0.136 fL	−0.329 to 0.058	0.166	0.703
Mean corpuscular haemoglobin	564	−0.055 pg	−0.129 to 0.020	0.150	0.703
Mean corpuscular haemoglobin concentration	564	−0.013 g/dL	−0.053 to 0.026	0.497	0.782
Leukocyte count	564	−0.704% relative	−1.656 to 0.258	0.148	0.703
Neutrophil count	564	−0.736% relative	−1.903 to 0.445	0.216	0.703
Lymphocyte count	564	−0.454% relative	−1.803 to 0.914	0.507	0.782
Platelet count	564	−0.126% relative	−1.039 to 0.795	0.785	0.785
Neutrophil to lymphocyte ratio	564	−0.283% relative	−1.848 to 1.306	0.721	0.782
Platelet to lymphocyte ratio	564	0.330% relative	−0.952 to 1.629	0.611	0.782

All models use Model 2 adjustment. BH correction is separate within the five-outcome vascular/metabolic and thirteen-outcome haematologic/red cell families. Native RDW and haematocrit changes are percentage points (pp). Natural-log cell count and ratio outcomes are expressed as relative percentage differences. Erythrocyte counts are in million/µL. CIs are pointwise, not FDR-adjusted. No endpoint met its within-family q < 0.05 threshold. Complete direct volume sensitivities are in Table S6.

## Data Availability

Aggregate results and measurement definitions are reported in the Supplementary Materials. Data-free analysis code, dated specifications, and full-precision aggregate tables are supplied separately for editorial and peer review in Non-published Material.zip. Reproducing participant-level models requires authorised access to the controlled study inputs and preparation specifications. Individual clinical and biomonitoring records, laboratory identifiers, and linkage files are subject to SPES privacy and institutional data-governance restrictions. Requests may be directed to the corresponding author, Carlo Buonerba (carbuone@hotmail.com), for consideration under the applicable institutional authorisation and data-use arrangements.

## Supplementary Materials

The following supporting information can be downloaded at https://www.mdpi.com/article/10.3390/ijms27219679/s1.
