# Supplementary material for: Plasma PAH-like AhR Bioactivity, Homocysteine, and Subclinical Vascular and Haematologic Phenotypes in the SPES Biomonitoring Substudy
Source: Int J Mol Sci. 2026 Oct 29;27(21):9679. doi: 10.3390/ijms27219679 (PMC13762868; doi:10.3390/ijms27219679)
Supplement: Supplementary file 1 [file ijms-27-09679-s001.zip › ijms-4634332-supplementary.pdf]

# Supplementary Material

**Plasma PAH-Like AhR Bioactivity, Homocysteine, and Subclinical Vascular and Haematologic Phenotypes in the SPES Biomonitoring Substudy**

Supplement to the research Article

This document provides supplementary methods, descriptive comparisons and complete results for the SPES PAH-CALUX analysis. Methods S1–S8 describe the original analyses; S9 and S10 describe revision-stage additions. References follow the numbering in the main manuscript. Full-precision aggregate tables, data-free code and dated specifications are supplied separately in Non-published Material.zip.

## Supplementary Methods

### S1. Analysis specification and sources

The internal statistical-analysis plan was frozen on 2 September 2026 before the initial association-calculation release. The corrected R implementation was executed on 4 September 2026. Subsequent implementation and reporting corrections preserved the primary exposure, endpoints and models. There was no external preregistration. The Akins amendment was prepared on 21 October and executed on 22 October 2026. The below-LOQ amendment was approved and executed on 24 October 2026. Both additions were made after the original results and reviewer comments were known and are explicitly post hoc.

Versioned source files linked assay records, clinical measurements and the participant crosswalk. The publication tables follow the completed R run; the pooled-model sex contrasts were recovered from its specified interaction models. Source, code and result checksums and the detailed verification records are retained in the author archive.

### S2. Participants and linkage

The clinical subgroup contained 600 participants. Two lacked an assay record and seven of the 598 PAH-CALUX results were below individual LOQs. The 591 quantified records contributed to the original primary and vascular/metabolic models. Twenty-seven lacked the entire CBC module; the haematologic models used 564 complete cases (Table S1). All 598 assay-record participants had complete primary outcomes and covariates and entered the post hoc below-LOQ sensitivities described in S10. Missing assays, outcomes, covariates and lipid measurements were never imputed.

The upstream laboratory file recorded participant identifier and blood-kit code together. Exact linkage produced 600 unique kit–participant pairs, corroborated by age, sex, sampling cluster and independently identifier-keyed clinical records. The raw seated-pressure field supplied the BP outcomes; source-specific absences in derived datasets were not treated as missing raw measurements.

The observed age range was 20–50 years, including two participants aged 50. Records included two current smokers, 37 reported antihypertensive users and a BMI range of 16.90–38.57 kg/m². Some BMI values exceeded the intended subgroup restriction. All observed eligible records were retained; the available selection records did not establish the reason for each departure from intended smoking, treatment and BMI criteria. A source-specific height discrepancy left the identity linkage intact, and the subgroup BMI field was retained.

### S3. PAH-CALUX procedure and available QC

#### S3.1. Human plasma processing and assay procedure

Following the official SPES sample-processing procedure, blood collected into lithium-heparin tubes was centrifuged at 2,500 × g for 10 minutes at +4 °C. Separated plasma was aliquoted and stored at −80 °C [27], then shipped frozen on dry ice to BDS. Routine clinical measurements followed the separate workflow described in S5. Aliquots of 0.5 g human plasma were extracted twice with 5 mL methyl-tert-butyl ether; upper layers were pooled, evaporated under nitrogen and reconstituted in 25 µL DMSO. Mean BaP extraction-and-cleanup recovery was 77%. No additional participant-specific recovery correction was applied in the epidemiological analysis. Serial extract dilutions were tested in technical triplicate in H4IIe rat hepatoma reporter cells for four hours at 0.8% DMSO. Luminescence was measured with a Berthold luminometer after luciferin addition (BDS, direct communication). The four-hour exposure and 0.8%

DMSO are supported by the BDS-reviewed method, supplied plate record and PAH-specific table in the official SPES report [27], despite differing entries in the 2019 BDS summary report.

### S3.2. Calibration, dilution selection and quantitative precision

Each 96-well plate included a complete BaP dose-response calibration curve; sample responses were interpolated using GraphPad Prism 5.03. High-response extracts were remeasured at additional dilutions to obtain responses in the linear range. The additional-dilution plate linked to the maximum result had recorded lower and upper response boundaries of approximately $1.8 \times 10^{-10}$ and $3.8 \times 10^{-9}$ mol/L. These describe that plate's response-acceptance window. Selected responses were marked acceptable in qualitative replicate-variability checks. Published assay-development repeatability and reproducibility were 5.9% and 6.6%, respectively [4]. Those validation estimates are reported separately from SPES control data. Technical triplicates were replicate wells, with participants forming the epidemiological units of analysis.

### S3.3. Procedure blanks and reference plasma

Procedure blanks and BaP-spiked human reference plasma accompanied the analytical series. Reference plasma was prepared by spiking with analytical-grade BaP, mixing overnight at room temperature and storing 0.5-mL aliquots at −20 °C. Ten reference aliquots were characterised by the bioassay. Blank and reference responses were monitored with control charts showing the mean and one- and three-standard-deviation lines. Blank responses were generally low, with one elevated blank followed by acceptable background in the next series. Reference-plasma bioactivity averaged approximately 23 ng BaP-EQ/mL. Most results were within the three-standard-deviation limits; elevated reference values were no longer observed after additional laboratory measures. BDS reported that overall PAH-CALUX performance fulfilled its QA criteria. These summaries concern the PAH-CALUX blank and reference-plasma controls (BDS, direct communication).

### S3.4. Quantification limits and censored results

Plate-based LOQs on the sample-equivalent scale ranged from 0.13 to 1.5 ng BaP-EQ/mL, with a representative value of approximately 0.31 ng/mL. The supplied conversion used well-based molar values, 0.8% DMSO, 25 µL extract, BaP molecular mass 252 g/mol and a sample-volume parameter of 0.5 mL. Individual reported limits governed censoring classification. The seven results were left-censored at 0.14, 0.25, 0.28, 0.33, 0.34, 0.39 and 0.41 ng BaP-EQ/mL (Table S16). They were excluded from the original quantified-exposure analysis (n=591). Their observed clinical characteristics are described in Table S11, and the additional substitution scenarios in S10 and Table S18. The laboratory-reported per-volume values were retained without introducing a new density correction or common blank-based exclusion threshold.

### S3.5. Upper-range dilution measurements

BDS supplied additional-dilution measurements for the plasma extract with the maximum reported bioactivity of 7,900 ng BaP-EQ/mL (Table S17). The 100-fold response exceeded the upper acceptance limit and was not selected; the responses marked selected fell within the recorded limits. Variation among dilution-corrected estimates warrants caution in interpreting the exact magnitude of this maximum. The prespecified highest-1% sensitivity excluded this observation and yielded similar findings for both primary outcomes (Table S5). Dilution fields and selection flags follow the BDS output (direct communication).

### S3.6. Scope of available analytical records

Numerical SPES-specific within-run and between-run precision estimates, the technical-replicate acceptance threshold, the complete calibration-standard list, fitted-curve equation, goodness-of-fit limits and complete raw-well data were unavailable for this analysis. Sample-specific cytotoxicity and response-interference data were also unavailable, as were participant-specific repeat-extraction and release decisions following control excursions. The recovery, control-chart and dilution information supplied by BDS and the published assay-validation results provide the available analytical support. Repeated participant bioactivity measurements were unavailable for estimating within-person temporal reliability.

## S4. Exposure units and lipid normalisation

Participant activity was laboratory-reported in ng BaP-EQ/mL plasma and retained at full precision without an additional plasma-density or recovery correction. BDS also reported gravimetric lipid measurements. The primary denominator followed the IZSM clinical component sum specified in the analysis plan, using serum total cholesterol (TC), triglycerides (TG) and phospholipids (PL), each in mg/dL; this calculation reproduced the recorded lipid field across all 598 assay records:

Lipid percentage (% w/v; g/100 mL) = (TC + TG + PL)/1000.

Lipid-normalised bioactivity (ng BaP-EQ/g estimated lipid) = volume-based bioactivity/(lipid percentage/100).

The primary exposure was log2 of the full-precision derived value. The component sum supplied the denominator throughout the primary and internally specified lipid-normalised sensitivity analyses; direct-volume log2 activity was the principal alternative scale. An additional post hoc Akins denominator is described in S9. The below-LOQ scenarios in S10 retain the historical component sum. The original convention preserves the archived SPES exposure definition, and its influence is assessed through these separately labelled comparisons.

Below-LOQ strings were classified as censored at their individual reporting limits. All 591 quantified values, including the maximum of 7,900 ng BaP-EQ/mL, remained in the primary models. The upper-tail sensitivity retained observations at or below the R type-7 99th percentile of the primary exposure (n=585). Only the post hoc scenarios in S10 assigned numerical values to the seven censored results.

## S5. Outcome and covariate definitions

Homocysteine was recorded in µmol/L and analysed on the natural-log scale. Clinical assessment and venepuncture belonged to the enrolment encounter. The published field procedure instructed participants to fast for at least eight hours, with a staff check before venepuncture [18]. Individual fasting duration, clock time and exact within-visit intervals were unavailable. The official SPES report describes fresh material allocated to routine biochemical and virological analyses within three hours of collection [27]. Complete homocysteine assay-platform, specimen-to-method and assay-specific processing records were unavailable for this analysis.

The seated-pressure field recorded SBP, DBP, heart rate and oxygen saturation. One seated recording was available per participant. Device model, resting time, cuff selection, repeated readings and timing relative to venepuncture were unavailable in the retained records. The outcome therefore represents the recorded seated value. No clinical diagnosis was inferred from one reading.

Pulse pressure was SBP minus DBP. Erythrocytes were displayed in millions/µL by dividing the source count per µL by $10^6$. NLR was neutrophils/lymphocytes, both in $10^3$/µL. PLR was (platelets/1000)/lymphocytes, converting the source platelet count to the same concentration scale. Ratios required positive observed lymphocyte counts.

Core adjustment used age, sex, BMI, current smoking and sampling-cluster code. Age was (age−35)/10; BMI was centred at the primary-population mean. Female sex and sampling cluster 9 were references. There were 11 sampling-cluster codes and 63 municipalities. Age-category sensitivities used <30, 30–39 and ≥40 years.

There were 37 explicit positive treatment responses, five explicit negatives, 547 negative hypertension histories with a blank follow-up treatment field classified as a structural skip, and two unavailable questionnaire records (Table S13). The follow-up question concerned treatment for high blood pressure after the physician-diagnosed hypertension question. Derived untreated status required an explicit negative history; missing history or an unavailable questionnaire remained unknown. Thus, 552 were classified as untreated and 589 had known derived status. This questionnaire-based classification may miss treatment for an unreported diagnosis or another indication. The original treatment-adjusted SBP model used n=589 and the untreated-only sensitivity n=552.

Creatinine was available for all 591 quantified participants. Circulating folate and vitamin B12 measurements were unavailable for this analysis. Diet, alcohol, occupation, socioeconomic circumstances and detailed medication information were outside the frozen primary adjustment set. The frozen specification recorded an anomalous repeated-value pattern in the derived alcohol variable and incomplete derivation QC for an occupational-exposure scalar. Residual nutritional, lifestyle, occupational and socioeconomic confounding is acknowledged.

## S6. Regression, covariance and multiplicity

With E=log2(lipid-normalised PAH-CALUX), Y was ln(homocysteine) or SBP in mmHg. The models were:

Model 1: Y = intercept + βE + (age−35)/10 + sex + sampling-cluster fixed effects + error.

Model 2 added centred BMI and current smoking. Both primary analyses used N=591; matrix rank including the intercept was K=14 for Model 1 and K=16 for Model 2. Models were unweighted. The two Model 2 exposure tests formed the primary family; Model 1 supplied design-adjusted context.

For municipality g, residual scores were Xg′ug. The covariance was the cluster sandwich based on summed outer products of these scores, multiplied by [G/(G−1)]×[(N−1)/(N−K)]. The primary analyses used G=63, with t62 inference. HC3 sensitivity intervals and tests instead used residual degrees of

freedom N−K=575. Random-intercept exposure-term inference used the degrees of freedom returned by nlme, also 575 for the reported fits.

For natural-log outcomes, effects and interval limits were transformed as 100[exp(β)−1], giving relative differences in conditional geometric means. Native-scale coefficients were mean differences; RDW and haematocrit differences were percentage points. The 95% CIs were pointwise, not simultaneous.

Holm adjustment covered the two primary Model 2 tests. Benjamini–Hochberg correction was applied separately within the five vascular/metabolic and thirteen haematologic/red-cell outcomes for each labelled exposure scale. Intervals are pointwise. Sensitivity, interaction and spline tests are secondary or exploratory. The original models used observed quantified exposures and complete outcomes/covariates. The three post hoc below-LOQ scenarios in S10 are the sole exception to the original exposure-substitution rule and leave the original multiplicity decisions unchanged.

## S7. Sensitivity and exploratory analyses

The internally specified sensitivities used direct-volume exposure, highest-1% exclusion, current-smoker exclusion, HC3 covariance and age categories. The smoking term was omitted in the nonsmoker-only subset (n=589). Clinical sensitivities added creatinine for homocysteine or adjusted for antihypertensive treatment (n=589) or restricted to those classified as untreated (n=552) for SBP (Table S5). The additional Akins and below-LOQ analyses are described separately in S9 and S10.

The pooled interaction models added E×sex to Model 2. The female-specific slope was βE and the male-specific slope was βE+βinteraction, with the covariance included in the variance of the sum. Both used t62 CIs. Formal interaction tests, rather than comparison of within-sex significance, assessed sex differences (Table S7).

Restricted cubic splines used the 10th, 50th and 90th percentiles of log2 exposure (Table S7b). The overall hypothesis tested both exposure terms using the two-degree-of-freedom robust F statistic with G−1 denominator degrees of freedom. Nonlinearity was assessed by the t test for the nonlinear component.

The geographic sensitivity used impact-area fixed effects and a sampling-cluster random intercept, fitted by maximum likelihood with nlme (Table S8). Cook's distance and externally studentised residuals describe influence in the primary Model 2; the 4/n cutoff was a diagnostic flag, not an exclusion rule (Table S9).

## S8. Software and reporting

Original analyses used R 4.5.1 (R Foundation for Statistical Computing, Vienna, Austria), 64-bit Windows; original package versions are retained in Table S10. The Akins and below-LOQ sensitivities used R 4.6.1 on Windows. The original models were reproduced against archived full-precision results before new fits, and source integrity, linkage, units, model rank, denominators and release privacy were checked. The LOQ run passed all 74 synthetic tests and ten release gates.

Data-free code, dated specifications and full-precision aggregate results are supplied separately in Non-published Material.zip. Participant-level inputs, identifiers, crosswalks and record-level diagnostics remain under institutional control. The publication tables below use display rounding. The original primary results and secondary hypothesis families were preserved throughout the revision.

### S9. Revision-stage descriptive comparisons and Akins sensitivity

These additions followed knowledge of the original results and reviewer comments. Tables S11 and S12 compare observed characteristics by quantification status and CBC availability. Table S13 distinguishes explicit treatment responses from questionnaire-derived negatives. Table S14 compares the quantified subgroup with available parent-study aggregates [27]; parent totals include the subgroup. Continuous summaries use medians and type-7 interquartile ranges, with observed denominators specified. These comparisons involved no formal group-comparison tests, selection weights or new CBC association models.

The additional Akins analysis was specified on 21 October and executed on 22 October 2026. The complete equation was TL (mg/dL) = 1.677(TC−FC)+FC+TG+PL, where TC is total cholesterol, FC free cholesterol, TG triglycerides and PL phospholipids [31]. All four serum components were measured. Volume-based PAH-CALUX was divided by TL/100000 (g/mL) and log2-transformed. Both primary-outcome models retained the original Model 2 covariates, finite-sample-adjusted municipality-clustered covariance and t62 pointwise intervals. All six historical, Akins and

volume-based comparator fits used the same 591 participants and 63 municipalities. The new analysis preserved the original primary tests and introduced no new confirmatory family (Table S15).

### S10. Post hoc below-LOQ substitution sensitivity

A dated amendment was approved on 24 October 2026 after the original findings and reviewer comments were known. It authorised deterministic substitution solely for the seven left-censored PAH-CALUX records in three additional sensitivity scenarios. Each result was assigned its own LOQ/√2, LOQ/2 or LOQ on the ng BaP-EQ/mL scale. The original component-sum lipid denominator and log2 transformation were then applied. All 591 measured positive values remained unchanged; the two participants without an assay record remained excluded. Outcomes, covariates and lipids were not imputed.

The six additional fits repeated Model 2 for natural-log homocysteine and recorded seated SBP. Each used n=598, 63 municipalities, model-matrix rank 16, the frozen BMI-centering mean and original factor coding. Finite-sample-adjusted municipality-clustered covariance and t62 inference were retained. Homocysteine estimates and interval limits were transformed as 100[exp(β)−1]; SBP was reported in mmHg. The original models were reproduced before the new fits, all individual LOQs and linkages were reconciled, and source checksums were unchanged after execution. Table S18 reports every scenario alongside the original results.

These scenarios evaluate sensitivity to three fixed assumptions about unquantified activity. Their pointwise intervals condition on the assigned values and omit uncertainty in the actual sub-LOQ measurements. The LOQ-boundary scenario limits the assumed censored exposure and does not provide a worst-case bound on the regression coefficient. The original quantified-only analysis remains primary; Holm and BH decisions are unchanged.

## Supplementary Results

The primary and vascular/metabolic populations contained 591 participants. CBC absence affected 11 of 24 quantified participants in sampling-cluster code 20 and 16 of 34 in code 21; the other contributing codes had complete CBC data. The haematologic models therefore used 564 participants (Table S3b).

Holm-adjusted p values were 0.699 for homocysteine and 0.183 for SBP. Vascular/metabolic q values were 0.417; haematologic/red-cell q values ranged from 0.703 to 0.785. Complete estimates, sensitivity analyses, exploratory contrasts and diagnostics are reported below.

## Supplementary Tables

Tables S1–S10 (including S3b, S3c and S7b) retain the original participant flow, definitions and results. Tables S11–S14 add descriptive comparisons and treatment-coding provenance; S15 reports the post hoc Akins sensitivity, S16–S17 the additional laboratory documentation, and S18 the post hoc below-LOQ sensitivity. Figures S1–S2 show the distributions of the quantified exposure.

**Table S1. Assay-specific participant flow.**

| Stage | n | Definition |
|---|---|---|
| Selected clinical subgroup | 600 | All kit-coded clinical records |
| CALUX assay roster | 598 | Two selected clinical records lack a CALUX assay record |
| Below-LOQ PAH records | 7 | Excluded from original quantified-only models; included by substitution in post hoc sensitivities (S10). |
| Quantified PAH analytical population | 591 | Both Tier 1 pairs and core model covariates complete |
| Vascular/metabolic Tier 2 population | 591 | All five outcomes complete |
| CBC/red-cell Tier 2 population | 564 | Whole CBC module unavailable for 27 quantified PAH participants |
| Post hoc below-LOQ sensitivity models | 598 | Includes all 591 quantified and seven censored records; two missing assays excluded. |

The primary population is defined by quantified PAH-CALUX and complete primary outcomes/covariates. CBC absence affects only the haematologic models. The original analysis excluded the seven censored results; the post hoc sensitivities include all 598 assay-record participants (S10; Table S18).

**Table S2. PAH-CALUX measurement and exposure derivation.**

| Item | Definition | Source / reporting basis |
|---|---|---|
| PAH extraction | 0.5 g plasma; two 5 mL methyl-tert-butyl ether extractions; final extract 25 µL DMSO | BDS laboratory methods (direct communication). |
| PAH assay | H4IIe cells; 0.8% DMSO; 4 h; triplicate serial dilutions; complete benzo[a]pyrene curve on each plate | BDS-reviewed method and supplied plate record; official SPES report, Table 7 [27]. See S3.1 for the differing entries in the 2019 BDS summary report. |
| Volume-based exposure | Laboratory-reported ng BaP-EQ/mL plasma; positive quantified values | Laboratory-reported assay result field; no additional density conversion applied. |
| IZSM lipid percentage | (Cholesterol + triglycerides + phospholipids, each mg/dL)/1000 | Clinical component-sum calculation; % w/v. |
| Primary exposure | log2[volume-based activity / (lipid percentage/100)] | ng BaP-EQ/g lipid; effect per exposure doubling. |

**Table S3. Continuous descriptives for the principal exposure, outcomes and context variables.**

| Variable/unit | n | Missing | Mean (SD) | Median (IQR) | Range |
|---|---|---|---|---|---|
| Age (years) | 591 | 0 | 34.50 (8.48) | 34.00 (27.00–42.00) | 20.00–50.00 |
| Body mass index (kg/m²) | 591 | 0 | 24.91 (3.62) | 24.44 (22.42–27.34) | 16.90–38.57 |
| PAH-CALUX, volume-based (ng BaP-EQ/mL) | 591 | 0 | 86.73 (385.85) | 27.00 (6.80–89.00) | 0.26–7,900.00 |
| PAH-CALUX, IZSM lipid-normalised (ng BaP-EQ/g lipid) | 591 | 0 | 16,375.32 (71,711.44) | 5,150.98 (1,199.66–16,211.22) | 47.27–1,364,421.42 |
| IZSM lipid-component sum (%) | 591 | 0 | 0.551 (0.117) | 0.534 (0.475–0.603) | 0.318–1.746 |
| Homocysteine (µmol/L) | 591 | 0 | 8.44 (1.96) | 8.09 (7.20–9.30) | 4.80–17.00 |
| Seated systolic blood pressure (mmHg) | 591 | 0 | 119.10 (12.96) | 120.00 (110.00–130.00) | 80.00–180.00 |
| Seated diastolic blood pressure (mmHg) | 591 | 0 | 76.08 (9.14) | 80.00 (70.00–80.00) | 50.00–110.00 |
| Heart rate (beats/min) | 591 | 0 | 73.69 (10.99) | 73.00 (66.00–80.00) | 44.00–110.00 |
| Creatinine (mg/dL) | 591 | 0 | 0.85 (0.16) | 0.80 (0.70–0.90) | 0.50–2.50 |
| Glucose (mg/dL) | 591 | 0 | 78.18 (8.37) | 77.00 (72.00–84.00) | 65.00–120.00 |
| Uric acid (mg/dL) | 591 | 0 | 4.82 (1.28) | 4.70 (3.90–5.70) | 1.60–9.60 |
| Red-cell distribution width (%) | 564 | 27 | 12.22 (0.92) | 12.04 (11.65–12.60) | 10.45–18.13 |

Values are rounded only for display; the CSV retains numerical precision. Untransformed PAH-CALUX means and SDs reflect the upper tail. Lipid percentage is % w/v; laboratory-reported volume units are retained.

**Table S3b. CBC missingness by sampling-cluster code within quantified PAH participants.**

| Cluster code | Quantified PAH n | Complete CBC n | Missing CBC n |
|---|---|---|---|
| 9 | 59 | 59 | 0 |
| 10 | 59 | 59 | 0 |
| 11 | 59 | 59 | 0 |
| 12 | 59 | 59 | 0 |
| 13 | 60 | 60 | 0 |
| 14 | 59 | 59 | 0 |
| 16 | 60 | 60 | 0 |
| 17 | 59 | 59 | 0 |
| 19 | 59 | 59 | 0 |
| 20 | 24 | 13 | 11 |
| 21 | 34 | 18 | 16 |

The complete CBC module is absent for 27 participants: 11 in code 20 and 16 in code 21. The 11 sampling-cluster codes are distinct from the 63 municipalities used for covariance clustering.

**Table S3c. Native-unit distributions of all prespecified secondary outcomes.**

| Outcome (unit) | n / missing | Mean ± SD | Median (IQR) | Range |
|---|---|---|---|---|
| Seated diastolic blood pressure (mmHg) | 591 / 0 | 76.08 ± 9.14 | 80.00 (70.00–80.00) | 50.00–110.00 |
| Pulse pressure (mmHg) | 591 / 0 | 43.03 ± 9.79 | 40.00 (40.00–50.00) | 14.00–100.00 |
| Heart rate (beats/min) | 591 / 0 | 73.69 ± 10.99 | 73.00 (66.00–80.00) | 44.00–110.00 |
| Uric acid (mg/dL) | 591 / 0 | 4.82 ± 1.28 | 4.70 (3.90–5.70) | 1.60–9.60 |
| Glucose (mg/dL) | 591 / 0 | 78.18 ± 8.37 | 77.00 (72.00–84.00) | 65.00–120.00 |
| RDW (%) | 564 / 27 | 12.22 ± 0.92 | 12.04 (11.65–12.60) | 10.45–18.13 |
| Haemoglobin (g/dL) | 564 / 27 | 13.98 ± 1.52 | 14.00 (12.90–15.07) | 8.98–20.01 |
| Haematocrit (%) | 564 / 27 | 41.09 ± 4.33 | 41.11 (38.05–44.06) | 26.99–58.12 |
| Erythrocyte count ($10^6$ cells/μL) | 564 / 27 | 4.878 ± 0.541 | 4.842 (4.511–5.208) | 3.682–7.120 |
| MCV (fL) | 564 / 27 | 84.48 ± 5.74 | 85.03 (82.42–87.29) | 57.36–104.90 |
| MCH (pg) | 564 / 27 | 28.77 ± 2.25 | 29.20 (28.23–30.00) | 17.78–33.90 |
| MCHC (g/dL) | 564 / 27 | 34.05 ± 1.25 | 34.20 (33.42–34.84) | 26.94–36.80 |
| Leukocyte count (cells/μL) | 564 / 27 | 6,408 ± 1,662 | 6,192 (5,241–7,383) | 2,791–13,320 |
| Neutrophil count ($10^3$ cells/μL) | 564 / 27 | 3.55 ± 1.24 | 3.36 (2.66–4.16) | 0.70–9.20 |
| Lymphocyte count ($10^3$ cells/μL) | 564 / 27 | 2.15 ± 0.64 | 2.05 (1.71–2.50) | 0.71–4.82 |
| Platelet count (cells/μL) | 564 / 27 | 230,437 ± 51,854 | 224,100 (196,200–259,525) | 92,430–451,600 |
| Neutrophil-to-lymphocyte ratio | 564 / 27 | 1.76 ± 0.74 | 1.62 (1.26–2.06) | 0.45–6.76 |
| Platelet-to-lymphocyte ratio | 564 / 27 | 114.63 ± 37.32 | 109.37 (89.40–133.36) | 48.19–322.50 |

Missingness is relative to the 591-person quantified-exposure population. CBC-derived outcomes have 564 observed values and 27 missing. Descriptive statistics use native measurement units regardless of regression transformation. Erythrocytes are in $10^6$ cells/μL; neutrophils and lymphocytes in $10^3$ cells/μL; leukocytes and platelets in cells/μL.

**Table S4. Statistical and measurement specification.**

| Component | Specification | Inference / detail |
|---|---|---|
| Population | 591 quantified PAH-CALUX records; 564 for CBC outcomes | One-to-one assay–clinical linkage; unweighted complete-case models. |
| Primary exposure | log2 IZSM lipid-normalised PAH-CALUX | Per two-fold higher activity; volume-based exposure is a sensitivity. |
| Primary outcomes | ln(homocysteine); SBP in mmHg | Relative geometric-mean difference and native-unit mean difference, respectively. |
| Model 1 | Exposure + (age−35)/10 + sex + sampling-cluster fixed effects | K=14 including intercept; contextual model. |
| Model 2 | Model 1 + centred BMI + current smoking | Primary model; K=16. Female sex and sampling cluster 9 are references. |
| Primary covariance | Municipality cluster sandwich × [G/(G−1)]×[(N−1)/(N−K)] | G=63; t62 for primary estimates and CIs. |
| Primary multiplicity | Holm across the two Model 2 exposure tests | Pointwise 95% CIs; two-sided α=0.05. |
| Secondary native outcomes | DBP, pulse pressure, heart rate, uric acid, glucose, RDW, haemoglobin, haematocrit, erythrocytes, MCV, MCH, MCHC | RDW/haematocrit differences are percentage points; erythrocytes displayed as $10^6$ cells/µL. |
| Secondary log outcomes | Leukocytes, neutrophils, lymphocytes, platelets, NLR, PLR | Relative differences in conditional geometric means. |
| Secondary multiplicity | BH within vascular/metabolic (5) and haematologic/red-cell (13), separately by exposure scale | Same Model 2 adjustment; no imputation. |
| Derived variables | Pulse pressure=SBP−DBP; NLR=neutrophils/lymphocytes; PLR=(platelets/1000)/lymphocytes | Source platelets are cells/µL; neutrophils and lymphocytes are $10^3$/µL. |
| Sensitivity subsets | Highest-1% exclusion n=585; non-smokers n=589 | Type-7 cutoff, retain ≤99th percentile; omit smoking only in all-non-smoker subset. |
| Clinical sensitivities | Add creatinine n=591; add antihypertensive treatment n=589; untreated-only SBP n=552 | Treatment status: 37 yes, 552 no, 2 unknown. |
| Age specification | Age categories <30, 30–39, ≥40 years | Upper category includes age 50. |
| HC3 sensitivity | Heteroscedasticity-consistent covariance with leverage correction | Residual df=N−K=575; not municipality-clustered. |
| Sex interactions | Pooled Model 2 plus exposure×sex | Linear-combination slopes, covariance included; t62 intervals. |
| Nonlinearity | Restricted cubic spline: 10th/50th/90th exposure percentiles | Global 2-df robust F; nonlinear t test; G−1 denominator df. |
| Geographic sensitivity | Impact-area fixed effects and sampling-cluster random intercept; ML in nlme | Exposure-term df=575 for the reported fits. |
| Influence | Cook's distance and externally studentised residuals | 4/n is a descriptive flag; no primary exclusion or winsorisation. |

The table summarises the exposure, adjustment, outcome scales and inferential procedures used in the original quantified-exposure analyses. Sampling-cluster fixed effects and municipality-clustered covariance represent distinct aspects of geographic structure. The additional post hoc Akins and below-LOQ specifications are described in S9 and S10.

**Table S5. Complete primary-endpoint sensitivity analyses.**

| Analysis | n | Difference | 95% CI | Raw p |
|---|---|---|---|---|
| Homocysteine (% relative difference) | | | | |
| Direct volume exposure | 591 | −0.12 | −0.76 to 0.52 | 0.701 |
| Exclude highest 1% | 585 | 0.02 | −0.68 to 0.73 | 0.956 |
| Exclude current smokers | 589 | −0.12 | −0.76 to 0.52 | 0.702 |
| HC3 inference | 591 | −0.12 | −0.78 to 0.53 | 0.709 |
| Age-band adjustment | 591 | −0.13 | −0.76 to 0.50 | 0.682 |
| Add creatinine | 591 | −0.08 | −0.72 to 0.57 | 0.813 |
| Systolic blood pressure (mmHg) | | | | |
| Direct volume exposure | 591 | −0.38 | −0.80 to 0.05 | 0.082 |
| Exclude highest 1% | 585 | −0.34 | −0.81 to 0.13 | 0.156 |
| Exclude current smokers | 589 | −0.37 | −0.80 to 0.06 | 0.089 |
| HC3 inference | 591 | −0.37 | −0.78 to 0.05 | 0.081 |
| Age-band adjustment | 591 | −0.40 | −0.82 to 0.02 | 0.063 |
| Add antihypertensive treatment | 589 | −0.27 | −0.69 to 0.15 | 0.203 |
| Exclude antihypertensive users | 552 | −0.28 | −0.70 to 0.14 | 0.190 |

Rows are original Model 2 variants with the indicated exposure, subset, covariate or covariance change. Highest-1% exclusion uses the primary exposure (n=585); nonsmoker models omit the constant smoking term. Municipality-clustered inference uses G−1 degrees of freedom; HC3 uses residual df=575. P values are exploratory sensitivity results. See Table S8 for the random-intercept model and Tables S15 and S18 for the additional post hoc analyses.

**Table S6. Complete secondary results and direct-volume exposure sensitivities.**

**Table S6. Vascular/metabolic | Primary IZSM lipid-normalised.**

| Outcome | n | Difference | 95% CI | Raw p | BH q |
|---|---|---|---|---|---|
| Seated diastolic blood pressure | 591 | −0.181 mmHg | −0.508 to 0.146 | 0.272 | 0.417 |
| Pulse pressure | 591 | −0.186 mmHg | −0.615 to 0.243 | 0.390 | 0.417 |
| Heart rate | 591 | −0.134 beats/min | −0.462 to 0.194 | 0.417 | 0.417 |
| Uric acid | 591 | −0.016 mg/dL | −0.047 to 0.014 | 0.292 | 0.417 |
| Glucose | 591 | 0.219 mg/dL | −0.070 to 0.508 | 0.135 | 0.417 |

**Table S6 (continued). Vascular/metabolic | Direct-volume sensitivity.**

| Outcome | n | Difference | 95% CI | Raw p | BH q |
|---|---|---|---|---|---|
| Seated diastolic blood pressure | 591 | −0.176 mmHg | −0.498 to 0.145 | 0.277 | 0.589 |
| Pulse pressure | 591 | −0.200 mmHg | −0.630 to 0.231 | 0.358 | 0.589 |
| Heart rate | 591 | −0.089 beats/min | −0.423 to 0.245 | 0.596 | 0.596 |
| Uric acid | 591 | −0.011 mg/dL | −0.043 to 0.020 | 0.471 | 0.589 |
| Glucose | 591 | 0.234 mg/dL | −0.048 to 0.516 | 0.102 | 0.511 |

Effects are per two-fold higher exposure on the indicated scale. Covariates match Model 2. BH adjustment is separate within each family and exposure scale. Native RDW and haematocrit are percentage points; transformed counts and ratios are relative percentage differences. CIs are pointwise.

**Table S6 (continued). Haematologic/red-cell | Primary IZSM lipid-normalised.**

| Outcome | n | Difference | 95% CI | Raw p | BH q |
|---|---|---|---|---|---|
| Red-cell distribution width | 564 | 0.017 pp | −0.018 to 0.052 | 0.334 | 0.782 |
| Haemoglobin | 564 | −0.011 g/dL | −0.059 to 0.036 | 0.637 | 0.782 |
| Haematocrit | 564 | −0.025 pp | −0.165 to 0.115 | 0.722 | 0.782 |
| Erythrocyte count | 564 | 0.006 million/uL | −0.008 to 0.021 | 0.387 | 0.782 |
| Mean corpuscular volume | 564 | −0.136 fL | −0.329 to 0.058 | 0.166 | 0.703 |
| Mean corpuscular haemoglobin | 564 | −0.055 pg | −0.129 to 0.020 | 0.150 | 0.703 |
| Mean corpuscular haemoglobin concentration | 564 | −0.013 g/dL | −0.053 to 0.026 | 0.497 | 0.782 |
| Leukocyte count | 564 | −0.704 % relative | −1.656 to 0.258 | 0.148 | 0.703 |
| Neutrophil count | 564 | −0.736 % relative | −1.903 to 0.445 | 0.216 | 0.703 |
| Lymphocyte count | 564 | −0.454 % relative | −1.803 to 0.914 | 0.507 | 0.782 |
| Platelet count | 564 | −0.126 % relative | −1.039 to 0.795 | 0.785 | 0.785 |
| Neutrophil-to-lymphocyte ratio | 564 | −0.283 % relative | −1.848 to 1.306 | 0.721 | 0.782 |
| Platelet-to-lymphocyte ratio | 564 | 0.330 % relative | −0.952 to 1.629 | 0.611 | 0.782 |

**Table S6 (continued). Haematologic/red-cell | Direct-volume sensitivity.**

| Outcome | n | Difference | 95% CI | Raw p | BH q |
|---|---|---|---|---|---|
| Red-cell distribution width | 564 | 0.017 pp | −0.018 to 0.052 | 0.331 | 0.716 |
| Haemoglobin | 564 | −0.005 g/dL | −0.053 to 0.042 | 0.827 | 0.977 |
| Haematocrit | 564 | −0.007 pp | −0.147 to 0.133 | 0.919 | 0.986 |
| Erythrocyte count | 564 | 0.008 million/uL | −0.006 to 0.023 | 0.249 | 0.716 |
| Mean corpuscular volume | 564 | −0.134 fL | −0.333 to 0.066 | 0.186 | 0.716 |
| Mean corpuscular haemoglobin | 564 | −0.054 pg | −0.132 to 0.023 | 0.166 | 0.716 |
| Mean corpuscular haemoglobin concentration | 564 | −0.014 g/dL | −0.054 to 0.026 | 0.500 | 0.923 |
| Leukocyte count | 564 | −0.577 % relative | −1.544 to 0.399 | 0.241 | 0.716 |
| Neutrophil count | 564 | −0.637 % relative | −1.831 to 0.571 | 0.294 | 0.716 |
| Lymphocyte count | 564 | −0.261 % relative | −1.646 to 1.144 | 0.710 | 0.923 |
| Platelet count | 564 | 0.008 % relative | −0.904 to 0.929 | 0.986 | 0.986 |
| Neutrophil-to-lymphocyte ratio | 564 | −0.377 % relative | −2.007 to 1.280 | 0.648 | 0.923 |
| Platelet-to-lymphocyte ratio | 564 | 0.270 % relative | −1.052 to 1.609 | 0.686 | 0.923 |

Effects are per two-fold higher exposure on the indicated scale. Covariates match Model 2. BH adjustment is separate within each family and exposure scale. Native RDW and haematocrit are percentage points; transformed counts and ratios are relative percentage differences. CIs are pointwise.

**Table S7. Exploratory sex-specific contrasts from pooled interaction models.**

| Outcome/unit | n | Women: effect (95% CI) | Men: effect (95% CI) | Interaction p |
|---|---|---|---|---|
| Homocysteine (% relative) | 591 | 0.46 (−0.52 to 1.44) | −0.74 (−1.56 to 0.10) | 0.059 |
| SBP (mmHg) | 591 | −0.64 (−1.08 to −0.20) | −0.08 (−0.82 to 0.66) | 0.198 |

Women n=295; men n=296. Contrasts use Model 2 plus exposure×sex, with t62 intervals. The interaction p value tests the difference between slopes. These are exploratory contrasts, not separate primary regressions.

**Table S7b. Restricted cubic-spline tests and fixed exposure knots.**

| Outcome | n | 10th / 50th / 90th log2 knots | Global p | Nonlinear p |
|---|---|---|---|---|
| Homocysteine | 591 | 8.901654 / 12.330630 / 14.796409 | 0.513 | 0.318 |
| SBP | 591 | 8.901654 / 12.330630 / 14.796409 | 0.124 | 0.837 |

The global test is a 2-df robust F test with G−1 denominator degrees of freedom. The nonlinear component uses a t test. Knots were fixed at the stated exposure percentiles.

**Table S8. Area-fixed, sampling-cluster-random-intercept sensitivity.**

| Outcome/unit | n | Difference (95% CI) | Raw p | Random-intercept variance |
|---|---|---|---|---|
| Homocysteine (% relative) | 591 | −0.15 (−0.78 to 0.49) | 0.645 | 0.013795 |
| SBP (mmHg) | 591 | −0.34 (−0.75 to 0.07) | 0.101 | 1.219386 |

Models were fitted by maximum likelihood with nlme. Exposure-term inference used the reported nlme degrees of freedom (575). Random-intercept variances are on the ln(homocysteine) and native-SBP outcome scales.

**Table S9. Aggregate primary-model influence diagnostics.**

| Endpoint | n | Maximum Cook's D | Number D > 4/n | Maximum \|studentised residual\| |
|---|---|---|---|---|
| Homocysteine | 591 | 0.0449 | 32 | 3.642 |
| Seated systolic blood pressure | 591 | 0.0410 | 25 | 5.002 |

Diagnostics describe the fitted primary Model 2. The 4/n threshold is a flag only. No primary observation was removed on the basis of these values, and participant identifiers are not included.

**Table S10. Software versions from the completed R analysis.**

| Software | Version |
|---|---|
| R | 4.5.1 (2025-06-13 ucrt), 64-bit Windows |
| digest | 0.6.39 |
| openxlsx | 4.2.8.1 |
| nlme | 3.1.168 |
| readxl | 1.4.5 |

These versions describe the original analysis. Additional Akins and below-LOQ runs used R 4.6.1, with their software records retained in the separate reproducibility releases.

**Table S11. Characteristics of quantified and below-LOQ participants.**

| Characteristic | Quantified (n=591) | Below LOQ (n=7) |
|---|---|---|
| Age (years) | 34.0 (27.0–42.0) | 43.0 (33.0–45.5) |
| BMI (kg/m²) | 24.44 (22.42–27.34) | 24.76 (22.59–27.89) |
| Homocysteine (µmol/L) | 8.09 (7.20–9.30) | 9.19 (7.86–10.50) |
| Seated SBP (mmHg) | 120.0 (110.0–130.0) | 120.0 (115.0–127.5) |
| Creatinine (mg/dL) | 0.80 (0.70–0.90) | 0.70 (0.70–0.85) |
| Lipid component sum (% w/v) | 0.534 (0.475–0.603) | 0.487 (0.445–0.514) |
| PAH-CALUX (ng BaP-EQ/mL) | 27.00 (6.80–89.00) | Below individual LOQ |
| PAH-CALUX (ng BaP-EQ/g estimated lipid) | 5150.98 (1199.66–16211.22) | Below individual LOQ |
| Female | 295/591 (49.9%) | 5/7 (71.4%) |
| Current smoker | 2/591 (0.3%) | 0/7 (0.0%) |
| High-impact area | 236/591 (39.9%) | 2/7 (28.6%) |
| Medium-impact area | 236/591 (39.9%) | 4/7 (57.1%) |
| Low-impact area | 119/591 (20.1%) | 1/7 (14.3%) |
| Antihypertensive treatment (coded yes) | 37/589 (6.3%) | 1/7 (14.3%) |

Continuous values are medians (IQRs); categorical values are n/available N (%). All listed non-treatment variables were observed in the relevant group, except the seven censored bioactivity results. These are below-LOQ results, not missing assay records. Two treatment records are unavailable in the quantified population. No group-comparison p values were calculated.

The seven below-LOQ records are separate from the two selected participants without an assay record. This descriptive comparison uses observed characteristics; the assigned exposure values used in the additional sensitivity scenarios are described in S10. Individual reporting limits are listed in Table S16.

**Table S12. CBC availability among quantified PAH-CALUX participants.**

| Characteristic | CBC available (n=564) | CBC unavailable (n=27) |
|---|---|---|
| Age (years) | 34.0 (27.0–42.0) | 34.0 (28.0–41.5) |
| BMI (kg/m²) | 24.41 (22.49–27.34) | 24.84 (20.83–27.55) |
| Homocysteine (µmol/L) | 7.98 (7.20–9.30) | 8.90 (7.65–10.10) |
| Seated SBP (mmHg) | 120.0 (110.0–126.5) | 120.0 (110.0–130.0) |
| Creatinine (mg/dL) | 0.80 (0.70–0.90) | 0.80 (0.70–0.90) |
| Lipid component sum (% w/v) | 0.536 (0.479–0.607) | 0.496 (0.438–0.560) |
| PAH-CALUX (ng BaP-EQ/mL) | 26.00 (6.35–85.00) | 39.00 (22.00–120.00) |
| PAH-CALUX (ng BaP-EQ/g estimated lipid) | 4793.38 (1171.48–15924.47) | 7865.17 (4208.83–23057.52) |
| Female | 281/564 (49.8%) | 14/27 (51.9%) |
| Current smoker | 2/564 (0.4%) | 0/27 (0.0%) |
| High-impact area | 236/564 (41.8%) | 0/27 (0.0%) |
| Medium-impact area | 209/564 (37.1%) | 27/27 (100.0%) |
| Low-impact area | 119/564 (21.1%) | 0/27 (0.0%) |
| Antihypertensive treatment (coded yes) | 34/562 (6.0%) | 3/27 (11.1%) |

Continuous values are medians (IQRs); categorical values are n/available N (%). All listed non-treatment variables were observed in both groups. Two treatment records are unavailable in the CBC-available group. All participants in this comparison have quantified PAH-CALUX results; the seven below-LOQ records are not included. No group-comparison p values were calculated.

CBC absence affected 11 of 24 quantified participants in sampling-cluster code 20 and 16 of 34 in code 21; all 27 belonged to medium-impact areas. The analysis of the thirteen haematologic outcomes uses complete cases (n=564). Possible selection associated with unobserved blood-count values remains a limitation.

**Table S13. Provenance of antihypertensive-treatment coding.**

| Source response / derivation | n | Analysis coding |
|---|---|---|
| Explicit treatment yes | 37 | Treated |
| Explicit treatment no | 5 | Untreated: explicit negative |
| Negative history; treatment structurally skipped | 547 | Untreated: derived negative |
| Questionnaire record unavailable | 2 | Unknown |

Total n=591: 37 treated, 552 untreated (five explicit and 547 derived negatives), and two unknown. The follow-up treatment item follows the physician-diagnosed hypertension question. A blank follow-up was classified as a structural skip only with an explicit negative history. Unavailable questionnaires or unresolved histories remained unknown. Reconstruction matched the frozen coding for all 591 participants. Treatment for another indication or an unreported diagnosis may be missed.

**Table S14. Contextual comparison with the parent SPES cohort.**

| Characteristic | Parent SPES (n=4,205) | Quantified PAH (n=591) |
|---|---|---|
| Participants | 4,205 | 591 |
| Age 20–29 years | 1,394 (33.2%) | 197 (33.3%) |
| Age 30–39 years | 1,296 (30.8%) | 198 (33.5%) |
| Age 40–50 years | 1,515 (36.0%) | 196 (33.2%) |
| High-impact area | 2,404 (57.2%) | 236 (39.9%) |
| Medium-impact area | 1,201 (28.6%) | 236 (39.9%) |
| Low-impact area | 600 (14.3%) | 119 (20.1%) |
| BMI, median (IQR), $kg/m^2$ | 24.6 (22.3–27.7) | 24.44 (22.42–27.34) |

Parent values are aggregate age, environmental-impact-area and BMI summaries from the official SPES report [27]. Parent totals include the subgroup. Counts and original rounding are retained; percentages may sum to slightly more or less than 100% after rounding. These descriptive comparisons do not estimate selection probabilities or the direction of selection bias. No selection weighting was performed.

**Table S15. Additional post hoc Akins lipid-normalisation sensitivity.**

| Exposure scale | n | Homocysteine<br>% difference (95% CI) | SBP<br>mmHg difference (95% CI) |
|---|---|---|---|
| Original component sum | 591 | −0.125 (−0.764 to 0.518) | −0.367 (−0.795 to 0.061) |
| Akins (post hoc) | 591 | −0.129 (−0.769 to 0.515) | −0.366 (−0.793 to 0.062) |
| Direct volume | 591 | −0.124 (−0.762 to 0.519) | −0.376 (−0.801 to 0.049) |

Effects are per two-fold higher bioactivity on the indicated scale. All six fits use the same 591 participants, 63 municipalities, original Model 2 covariates, matrix rank 16 and t62 inference. Confidence intervals are pointwise. Homocysteine was analysed on its natural-log scale and transformed to relative geometric-mean differences; SBP was analysed in mmHg. The additional Akins analysis leaves the original primary exposure and multiplicity decisions unchanged.

Complete Akins equation: TL (mg/dL) = 1.677(TC−FC)+FC+TG+PL [31]. All four serum components were measured; PAH-CALUX used human plasma. Volume-based bioactivity was divided by TL/100000 (g/mL) and log2-transformed. No exposure or lipid component was imputed. Results are from the completed R run of 22 October 2026.

**Table S16. Individual below-LOQ reporting limits.**

| Reported activity (ng BaP-EQ/mL) | Number of human plasma samples |
|---|---|
| <0.14 | 1 |
| <0.25 | 1 |
| <0.28 | 1 |
| <0.33 | 1 |
| <0.34 | 1 |
| <0.39 | 1 |
| <0.41 | 1 |

Each row identifies the reporting limit for one left-censored human plasma result. These seven records were excluded from the original quantified-exposure models (n=591) and included under the fixed assumptions in S10 and Table S18. Data courtesy of BioDetection Systems (direct communication and original analytical reports).

**Table S17. Additional-dilution records for the maximum reported bioactivity.**

| Laboratory dilution field | Well response (mol/L) | BDS selection / range flag |
|---|---|---|
| 100 | $8.6 \times 10^{-9}$ | No; above upper boundary |
| 1,000 | $2.2 \times 10^{-9}$ | Yes; within boundaries |
| 10,000 | $5.3 \times 10^{-10}$ | Yes; within boundaries |
| 100,000 | $4.7 \times 10^{-10}$ | Yes; within boundaries |
| 1,000,000 | $4.4 \times 10^{-10}$ | Yes; within boundaries |

Additional measurements concern the plasma extract with the maximum reported bioactivity of 7,900 ng BaP-EQ/mL. Selection flags and response limits are reproduced from the BDS output; the plate limits were approximately $1.8 \times 10^{-10}$ to $3.8 \times 10^{-9}$ mol/L. Dilution-corrected estimates varied across the tested dilutions, introducing uncertainty around the exact maximum. This observation was excluded in the prespecified highest-1% sensitivity (Table S5). Data courtesy of BioDetection Systems (direct communication).

**Table S18. Post hoc below-LOQ substitution sensitivity for the two primary outcomes.**

| Exposure handling | n | Homocysteine % difference (95% CI) | SBP mmHg difference (95% CI) |
|---|---|---|---|
| Original quantified-only | 591 | −0.125 (−0.764 to 0.518) | −0.367 (−0.795 to 0.061) |
| Individual LOQ/√2 | 598 | −0.211 (−0.806 to 0.387) | −0.323 (−0.699 to 0.053) |
| Individual LOQ/2 | 598 | −0.215 (−0.805 to 0.378) | −0.319 (−0.690 to 0.052) |
| Individual LOQ boundary | 598 | −0.207 (−0.806 to 0.396) | −0.328 (−0.708 to 0.053) |

Effects are per two-fold higher original component-sum lipid-normalised PAH-CALUX bioactivity. All fits use the original Model 2 covariates, 63 municipalities, matrix rank 16 and t62 municipality-clustered inference. The seven individual LOQs were substituted on the volume-based scale before lipid normalisation. All 591 quantified values remained unchanged and all 598 assay-record participants had complete outcomes and covariates. The two participants without an assay record remained excluded.

Intervals are pointwise and conditional on the assigned exposure values; they omit uncertainty in the actual sub-LOQ measurements. The LOQ boundary limits assumed censored activity and does not bound the regression coefficient. The six additional fits were executed on 24 October 2026 under an approved post hoc amendment. The original primary analysis and its Holm correction remain unchanged. All scenarios are shown irrespective of their results.

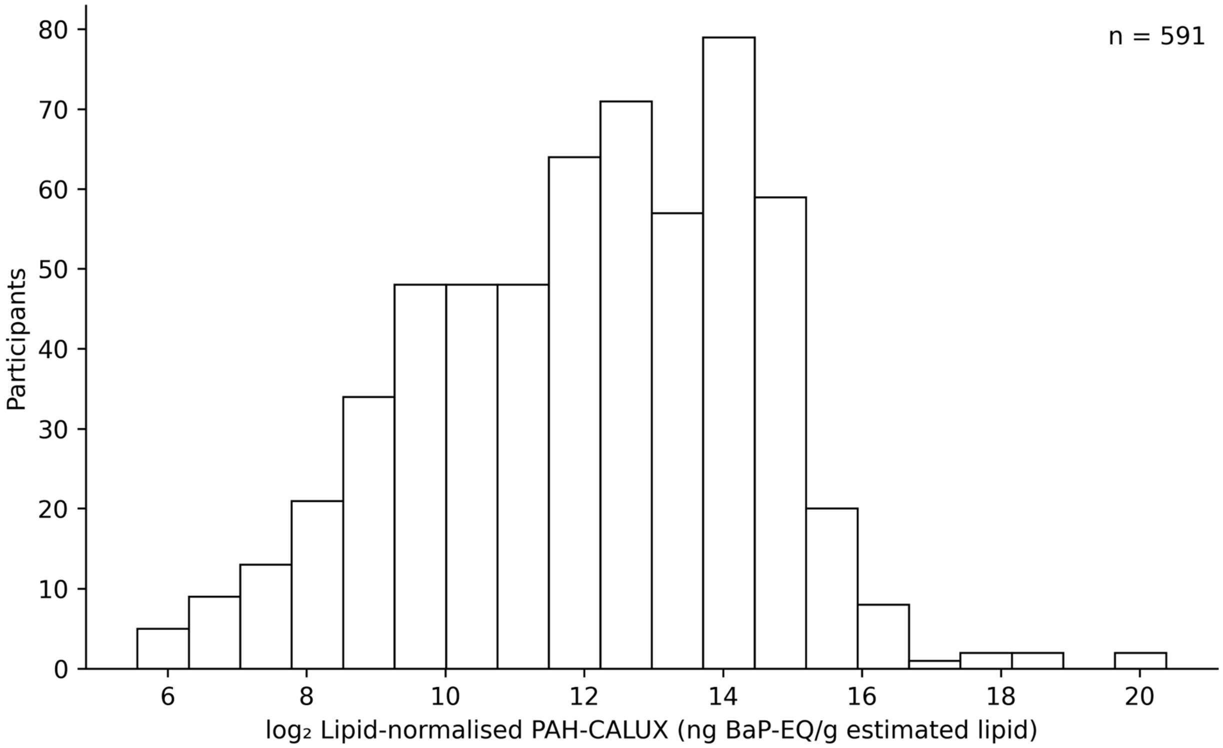


**Figure S1. Distribution of log2 PAH-CALUX normalised to the original clinical lipid-component sum (ng BaP-EQ/g estimated lipid). All 591 quantified measurements are included, without winsorisation. The seven below-LOQ results are analysed separately in the post hoc substitution scenarios and are omitted from this distribution.**

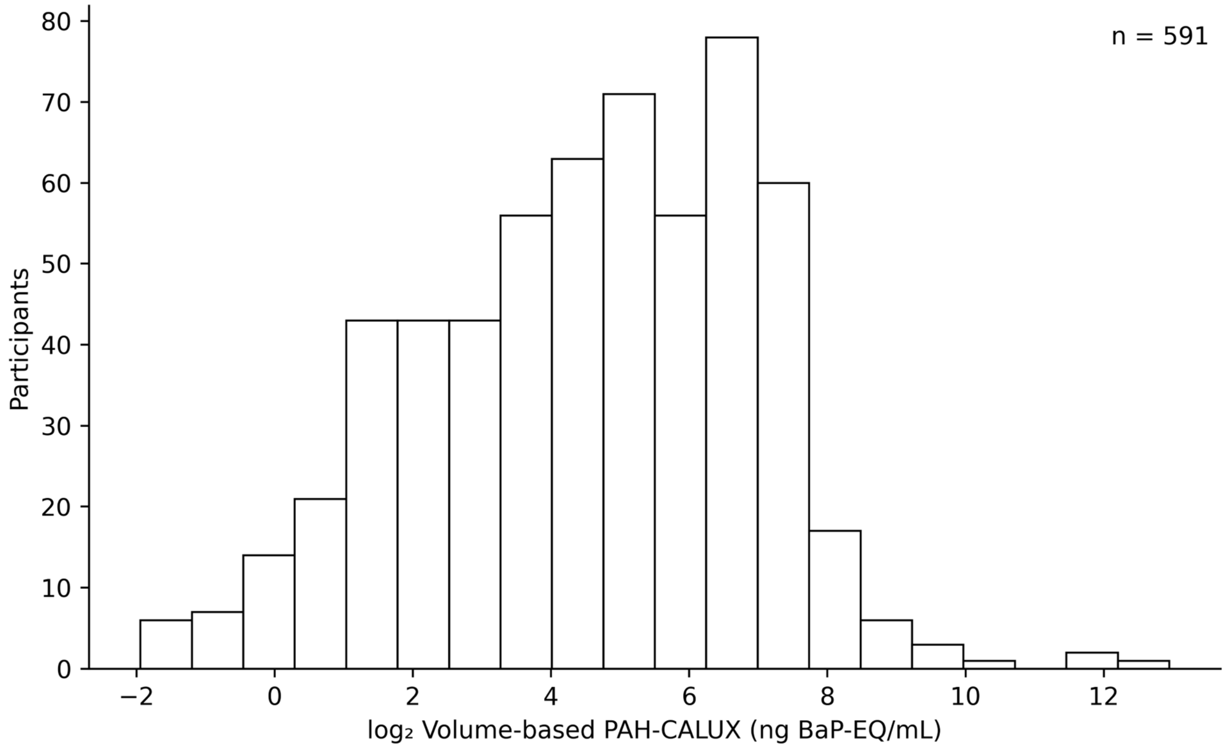


**Figure S2. Distribution of log2 laboratory-reported volume-based PAH-CALUX (ng BaP-EQ/mL). All 591 quantified measurements are included, without winsorisation. The seven below-LOQ results are analysed separately in the post hoc substitution scenarios and are omitted from this distribution.**
